# Supplementary material for: Enhanced quantum state transfer by circumventing quantum chaotic behavior
Source: Nat Commun. 2024 Jun 10;15:4918. doi: 10.1038/s41467-024-48791-3 (PMC11164980; doi:10.1038/s41467-024-48791-3)
Supplement: Supplementary file 1 — Supplementary Information [file 41467_2024_48791_MOESM1_ESM.pdf]

Supplementary Information for
Enhanced quantum state transfer by Circumventing quantum chaotic behavior

CONTENTS

Supplementary Note 1 - Device information 1

Supplementary Note 2 - Measurements of coupling strengths 1

Supplementary Note 3 - Effects of residual thermal populations 2

Supplementary Note 4 - Noise analysis for quantum state transfer 4

Supplementary Note 5 - Measurement of trajectory in large-spin representation 5

Supplementary Note 6 - Preparation of Bell state 9

Supplementary Note 7 - (Non)Ergodicity of two-excitation Hamiltonians 9

Supplementary Note 8 - Numerically optimized quantum state transfer with Monte Carlo annealing 12

Supplementary Note 9 - Quantum speed limit bounds 14

Supplementary Note 10 - Optimization solutions for much larger system sizes 14

Supplementary Note 11 - Comparison with sequential gate scheme 15

References 18

SUPPLEMENTARY NOTE 1 - DEVICE INFORMATION

A two-dimensional (2D) flip-chip superconducting quantum processor (Supplementary Figure 1) is utilized for our quantum state transfer (QST) experiments, which includes 36 qubits and 60 couplers. These qubits are built as a 6×6 square lattice, where a tunable coupler connects each nearest-neighbor (NN) qubit pair. Each qubit has an individual control line for single-qubit rotations and frequency modulation via applying microwave (XY) pulses and flux (fast Z and slow Z) pulses. Each coupler also has a control line for tuning the coupling strength of the connected qubit pair by applying the flux pulses. Details of experimental setup for control electronics and wiring are shown in Supplementary Figure 1.

Up to 36 qubits and 59 couplers are dynamically controlled during the experiments since the coupler between Q_{18} and Q_{24} malfunctions and presents limited control. Supplementary Figures 2 and 3 display typical performance for all 36 qubits, such as qubit idle frequency ω_{10}, energy relaxation time T_1, spin-echo dephasing time T_2^{SE}, and readout fidelities F_0 and F_1. Notably, the average energy relaxation time T_1 near interaction frequency ($\omega_I/2\pi = 4.585$ GHz) is above 140 μs (see Supplementary Figures 2**b** and **e**), and the spin-echo dephasing time T_2^{SE} has the mean value of ~ 19 μs (see Supplementary Figures 2**c** and **f**). Readout fidelities (see Supplementary Figure 3) are measured individually for each qubit, assisted with a reset protocol to reduce thermal populations. The average fidelity of single-qubit gates characterized by randomized benchmarking is around 0.999 in our experiments.

SUPPLEMENTARY NOTE 2 - MEASUREMENTS OF COUPLING STRENGTHS

In our protocol, the nearest-neighbor couplings and the residual cross-couplings provide channels for quantum state transfer, which makes their accurate measurements fundamental for accomplishing the desired effects. The coupling strength between each qubit pair is obtained via resonant photon swap dynamics. For each NN qubit pair, we measure its coupling strength twice, with all other qubits detuned $\pm\Delta$ away from the target interaction frequency (ω_I), and all other NN couplings kept at the same values as those in the QST process. Then, we can estimate the absolute value of the coupling strength by averaging over the two

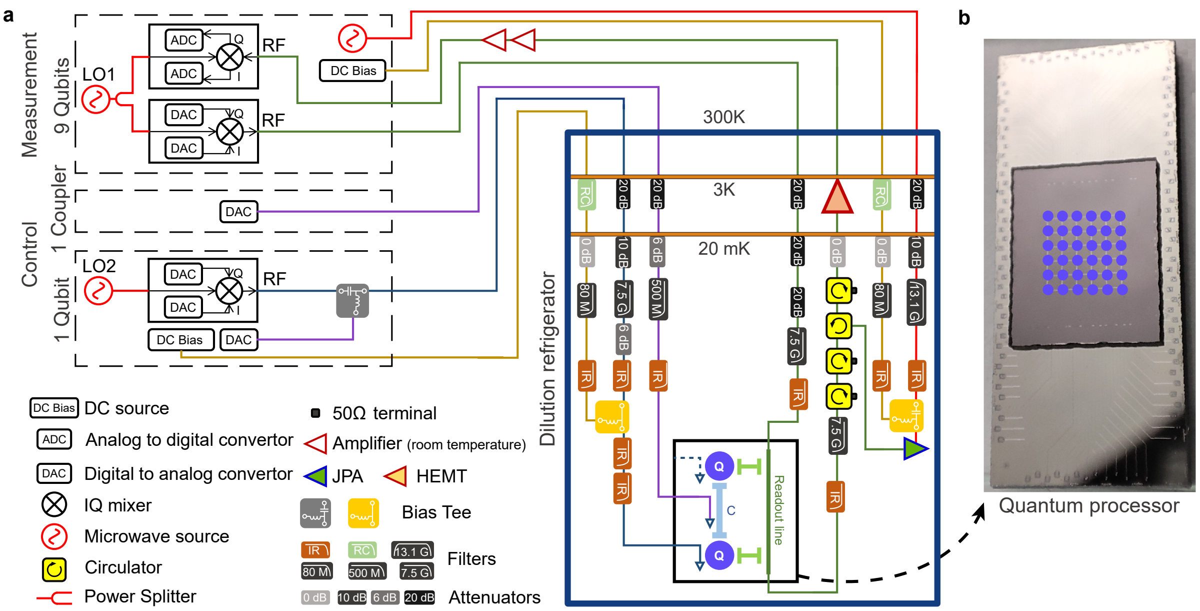

Supplementary Figure 1. **Experimental setup and quantum processor.** **a**, Illustration of room-temperature electronics and chip wiring. The qubits on the processor are divided into four readout groups. Each group consists of nine qubits that can be measured simultaneously using a common readout line. Readout signals are generated by two DAC channels, which are mixed with a local oscillator signal and then carried to the quantum processor. The returning readout signals are amplified by a Josephson parametric amplifier (JPA), a high electron mobility transistor (HEMT), and two room-temperature amplifiers before demodulated by the ADCs for qubit state discrimination. A fast flux (Z) signal is generated by a single DAC channel while a slow DC flux signal is generated by a DC source. Similar to the readout signal, a microwave (XY) pulse is generated by two DAC channels and further mixed with a local oscillator signal with an IQ mixer. We use a room-temperature Bias Tee to combine Z and XY pulses and a low-temperature Bias Tee to further combine them with a slow flux signal. **b**, A photograph of the flip-chip quantum processor. It accommodates a lattice of 6×6 qubits with nearest-neighbor tunable couplers.

measurement results. Since NN couplings are dominated by the virtual photon exchange mediated by the coupler, in the regime we consider, if the coupler's frequency is above the interaction frequency, the sign is negative; otherwise, it is positive.

The measurements are similar but relatively more complex for cross-coupling $J^\times$. We find that the measured coupling, labeled by $J(\Delta)$, depends on both NN coupling configurations and the frequencies of other qubits. Therefore, we measure $J(\Delta)$ for a set of detunings $\{\Delta_i\}$, such as $\Delta_i/2\pi = -150$ MHz, -120 MHz, -100 MHz, 150 MHz, and then estimate $J^\times$'s value by fitting the results with an approximate formula $J(\Delta) = J^\times + \mathrm{sign}(g)\frac{2g^2}{\Delta}$. Here, g is a factor representing the effective averaged NN couplings in the same plaquette, and $J^\times$ is the cross-coupling we want to measure. Physically, $J^\times$ is mainly caused by a parasitic capacitor directly connecting the two diagonal qubits in the plaquette. In addition, the weak coupling between the qubit and its next NN coupler also leads to a small contribution to $J^\times$ via a virtual photon interaction. Experimentally measured couplings for different cases of QST highlighted in the main text are shown in Supplementary Figure 4 (single-excitation QST in 1×6 and 3×3 networks), Supplementary Figure 5 (single-excitation QST in 6×6 network), Supplementary Figure 6 (Bell state QST in 6×6 network), Supplementary Figure 7 (two-excitation QST in 3×3 network), and Supplementary Figure 8 (two-excitation QST in 6×6 network). The transfer dynamics using the measured couplings are shown in Figs. 2, 3, and 4 of the main text. The final state populations after QST for them are shown in Supplementary Figure 9.

SUPPLEMENTARY NOTE 3 - EFFECTS OF RESIDUAL THERMAL POPULATIONS

In both the standard theoretical protocol [1] and our Monte Carlo annealing process, the underlying assumption is that the medium for transferring quantum information is always in its ground state, where no unwanted excitation exists. However, this is not true for real-world superconducting quantum devices. Despite being mounted in a dilution refrigerator with a base temperature of ~20 mK [2], spurious excitations often occur in the qubits [3] due to the black-body radiation from higher temperature parts or the thermal heating by control signals. In this section, we investigate the infidelity of quantum state transfer induced by residual thermal populations in the device.

Since a 1D chain with nearest-neighbor XY couplings is always an integrable Hamiltonian irrespective of the population, QST across the chain is insensitive to small thermal populations, as has been reported in Ref. [4]. The situation is different for the 2D XY model with cross-couplings that we explore. This system is chaotic in the presence of many excitations and therefore tends to suppress the QST once unwanted thermal populations arise. To quantify such effects on large 2D networks, we numerically

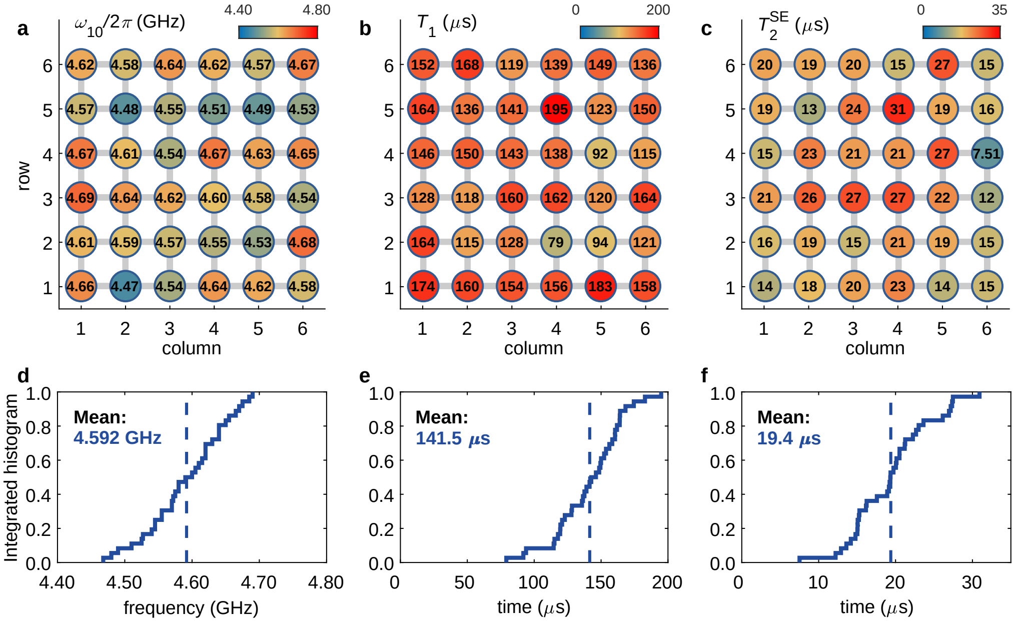

Supplementary Figure 2. **Coherence time for 36 qubits. a - c**, Heat map for typical values of idle frequency ω_{10} (**a**), energy relaxation time T_1 (**b**), and spin-echo dephasing time $T_2^{\rm SE}$ (**c**). T_1 and $T_2^{\rm SE}$ are measured near interaction frequency $\omega_I/2\pi = 4.585$ GHz where quantum state transfer happens. **d - f**, Histogram statistics of the corresponding results from **a - c**, where the mean value of each panel is highlighted.

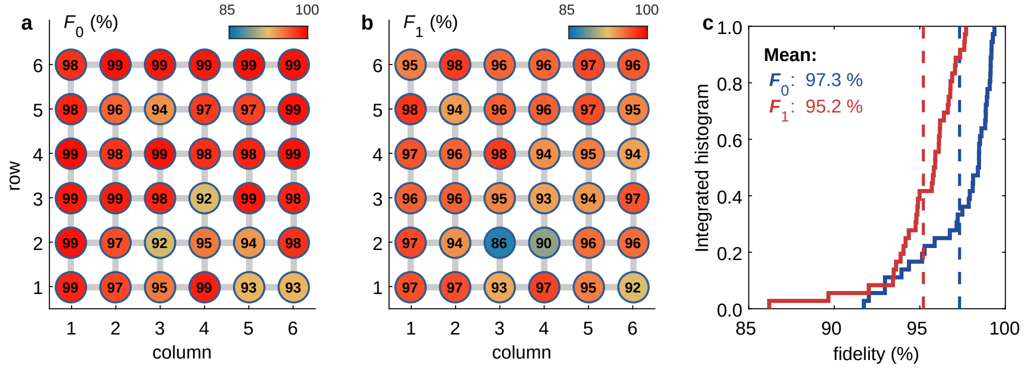

Supplementary Figure 3. **Readout performance for 36 qubits. a**, Heat map of readout fidelity F_0, which is obtained by preparing all the qubits in their ground states and measuring the probability of $|0\rangle$ state for each qubit. **b**, Heat map of readout fidelity F_1. Each value is measured by only exciting the target qubit and detecting the corresponding $|1\rangle$ state probability. **c**, Histogram statistics of F_0 and F_1, where the mean value of each panel is highlighted.

simulate small networks of various system sizes and then scale the results to large systems beyond the reach of our current classical computational power. For simplicity, we model the residual thermal population γ by setting the state of each qubit as $|\phi_0^i\rangle = \sqrt{1-\gamma_i}|0\rangle + e^{i\theta_i}\sqrt{\gamma_i}|1\rangle$ ($\sqrt{\gamma_i}|0\rangle + e^{i\theta_i}\sqrt{1-\gamma_i}|1\rangle$) for qubit Q_i expected to be $|0\rangle$ ($|1\rangle$) in initial state. γ_i is randomly sampled from a Gaussian distribution of $N(\gamma,\ 0.2\gamma)$ and θ_i is a random phase uniformly sampled from 0 to 2π. The Bell state in qubit pair (Q_1, Q_2) is generated by applying an ideal state-preparation quantum circuit (see Supplementary Figure 14**a**) to the ground state with thermal populations $\bigotimes_{i=1}^{2}\left(\sqrt{1-\gamma_i}|0\rangle + e^{i\theta_i}\sqrt{\gamma_i}|1\rangle\right)$. Figure 10**a** shows the population dynamics of single-excitation QST in the 3×3 network for $\gamma \in \{0, 0.5\%, 1.0\%, 2.0\%\}$. As expected, the fidelity of QST is significantly compromised as γ increases.

We further investigate the effects of residual thermal populations for different system sizes. Supplementary Figures 10**b**, **c**, and **d** show the numerical results of the infidelity caused by thermal population γ as a function of system size. For all three cases (single-excitation, Bell state, and two-excitation), the infidelity grows as the system size increases. Here, the infidelity is defined by $(F_{\gamma=0} - F_\gamma)/F_{\gamma=0}$, where F_γ is the transfer fidelity with thermal population of γ. To obtain the influence of thermal populations on QST in large systems, such as a 6×6 network, which cannot be simulated efficiently with classical computers, we perform a system size extrapolation by fitting the relation between the infidelity and the system size using the results of a small number of qubits. As a result, we can approximately estimate the infidelity caused by thermal populations for our experiments, even in the case of 36 qubits. As the typical value of residual thermal population after initialization is $\sim 0.5\%$ (see Supplementary

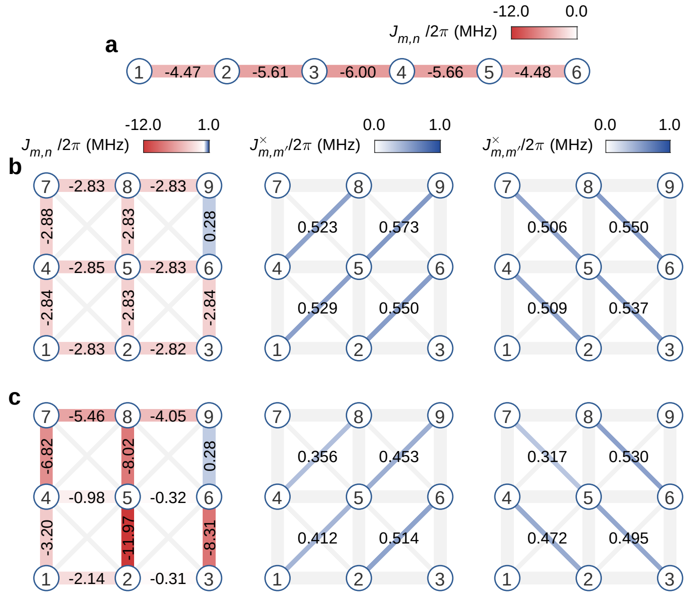

Supplementary Figure 4. **Experimentally measured couplings for single-excitation QST in small systems.** **a**, Measured couplings for single-excitation QST on a 6-qubit 1D chain. This coupling configuration is the standard 1D protocol of Ref. [1] and the corresponding dynamics are shown in Figs. 2**b** and **c** of the main text. **b**, Measured couplings for a 3×3 network with NN couplings parameterized using the standard protocol of Ref. [1]. In the ideal case, all the NN couplings are expected to be homogeneous for the 3×3 network, but due to the defect between Q_6 and Q_9, this coupling is fixed with $J_{\text{defect}}/2\pi \approx 0.3$ MHz. The corresponding transfer dynamics are shown in Figs. 2**e** and 2**f** of the main text. **c**, Measured couplings optimized with largely successful QST (see main text, Figs. 2**h** and **i**).

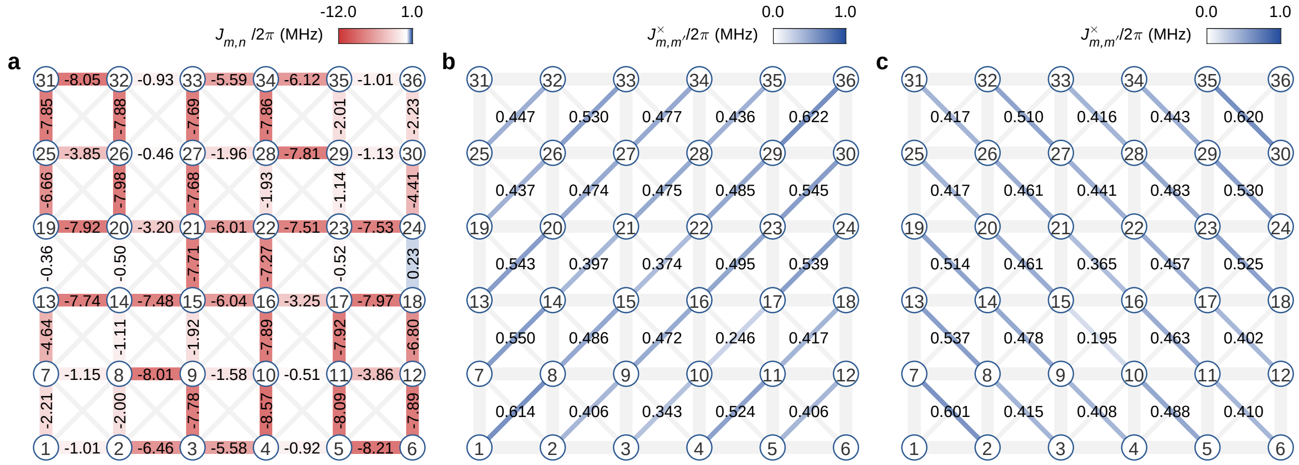

Supplementary Figure 5. **Experimentally measured couplings for single-excitation QST in the** 6×6 **network.** **a**, Measured NN couplings including the defect between Q_{18} and Q_{24}, utilizing an annealing optimized solution as target – see Fig. 3**a** in the main text. The defective coupling is about 0.2 MHz. **b** and **c**, Measured cross couplings.

Figure 11) for our system, we can deduce that the thermal population induced infidelity of QST in the 6×6 network is $\sim 10\%$.

SUPPLEMENTARY NOTE 4 - NOISE ANALYSIS FOR QUANTUM STATE TRANSFER

On top of the effects of thermal populations, other influences [6] can also affect the experimental realization of an efficient QST, including imperfect experimental control on couplings and qubit frequencies. To account for these, we numerically explore these effects on different cases of QST (see Supplementary Figure 12). We assume a Gaussian-distributed noise $\delta \sim N(0, \sigma)$, where σ is the standard deviation. For the noise on the originally optimized NN couplings $\{J_{m,n}\}$, we sample $\{\delta_{m,n}\}$ from $N(0, \sigma)$ and set the noise-affected NN couplings as $\{(1 + \delta_{m,n})J_{m,n}\}$. By averaging the numerical results over 200 instances, we show that the simulated QST fidelities decay as a function of the standard deviation σ in Supplementary Figure 12**a** for all cases considered

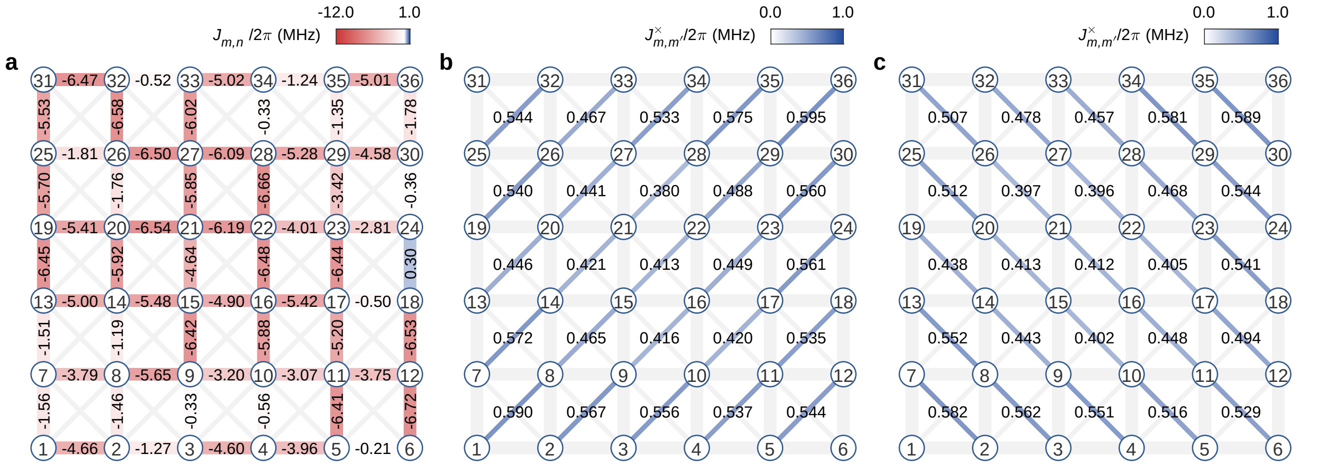

Supplementary Figure 6. **Experimentally measured couplings for Bell state QST in the** 6×6 **network. a**, Measured NN couplings including the defect between Q_{18} and Q_{24}, utilizing an annealing optimized solution as target – see Fig. 3**b** in the main text. The defective coupling is about 0.3 MHz. **b** and **c**, Measured cross-couplings.

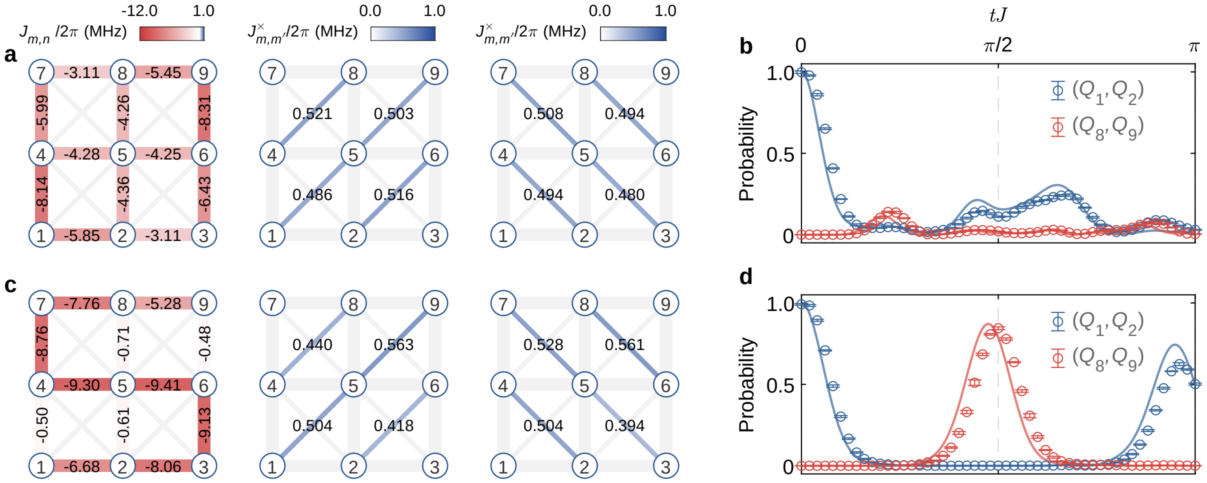

Supplementary Figure 7. **Experimentally measured couplings for two-excitation QST in the** 3×3 **network. a**, Measured NN and cross-couplings for the two-excitation QST in a 3×3 network with randomly selected NN couplings. **b**, Experimentally measured population dynamics (markers) and numerical results (lines) using the random coupling configuration in **a**. **c**, Measured NN and cross-couplings for the enhanced two-excitation QST in a 3×3 network with the annealing optimized NN couplings. **d**, Experimentally measured population dynamics (markers) and numerical results (lines) with the optimized NN couplings in **c**. The corresponding experimental results for the dynamics in Fock space are shown in Figs. 4**b** and **c** of the main text.

in the main text. As expected, the noise more easily affects the two-excitation case since it is on the verge of decaying to quantum chaotic behavior. This analysis demonstrates that the solutions for the couplings optimized by the annealing process are rather special.

For the case of noise in the qubit's frequency, the Hamiltonian is modified by adding diagonal terms of the form $\sum \hat{\sigma}_i^+ \hat{\sigma}_i^- \delta_i$. Here, σ_i^+ and σ_i^- are raising and lowering operators for the i-th qubit. For a given standard deviation σ, frequency disorders $\{\delta_i\}$ are randomly selected. By averaging over 200 such instances, we obtain the corresponding fidelities of the target qubit(s) (see Supplementary Figure 12**b**). Similar to the noise in the couplings, the QST-fidelity of the two-excitation case is quickly compromised for large σ.

SUPPLEMENTARY NOTE 5 - MEASUREMENT OF TRAJECTORY IN LARGE-SPIN REPRESENTATION

In the main text, we map single-excitation QST in a 2D network to the precession of two large spins under magnetic fields. We experimentally probe the dynamical trajectories of the two large spins by measuring their projections in the x, y, z directions of the Bloch sphere. Taking single-excitation QST in a 3×3 network as an example (Figs. 2**f** and **i** of the main text), the matrix

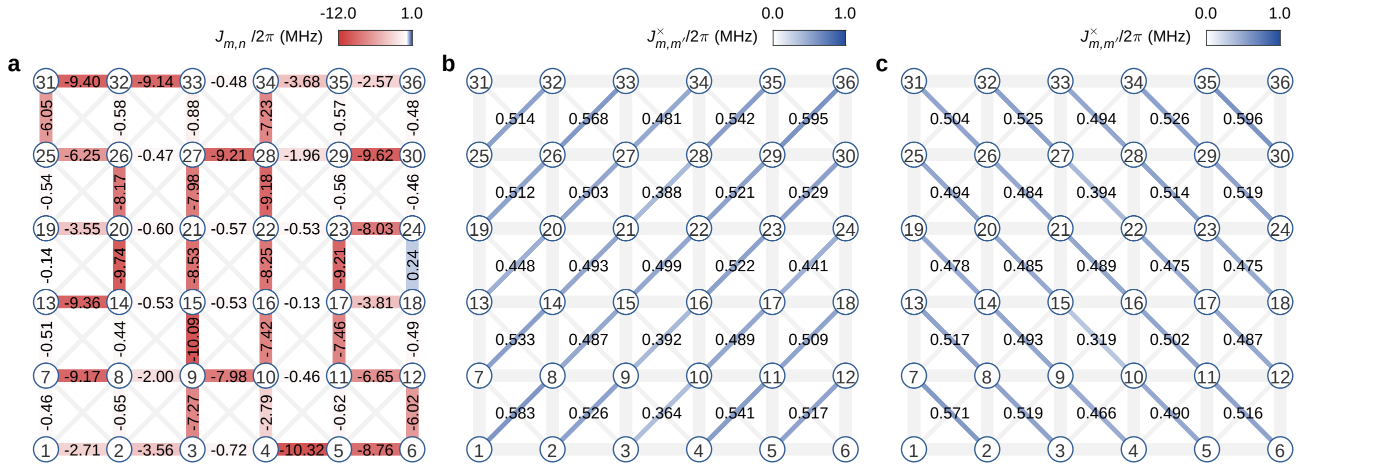

Supplementary Figure 8. **Experimentally measured couplings for two-excitation QST in the** 6×6 **network.** **a**, Measured NN couplings. The defect between Q_{18} and Q_{24} is $J_{\text{defect}}/2\pi \approx 0.2$ MHz. **b** and **c**, Measured cross-couplings. The corresponding experimental dynamics for transferring two excitations from (Q_1, Q_2) to (Q_{35}, Q_{36}) are shown in Fig. 4**e** of the main text.

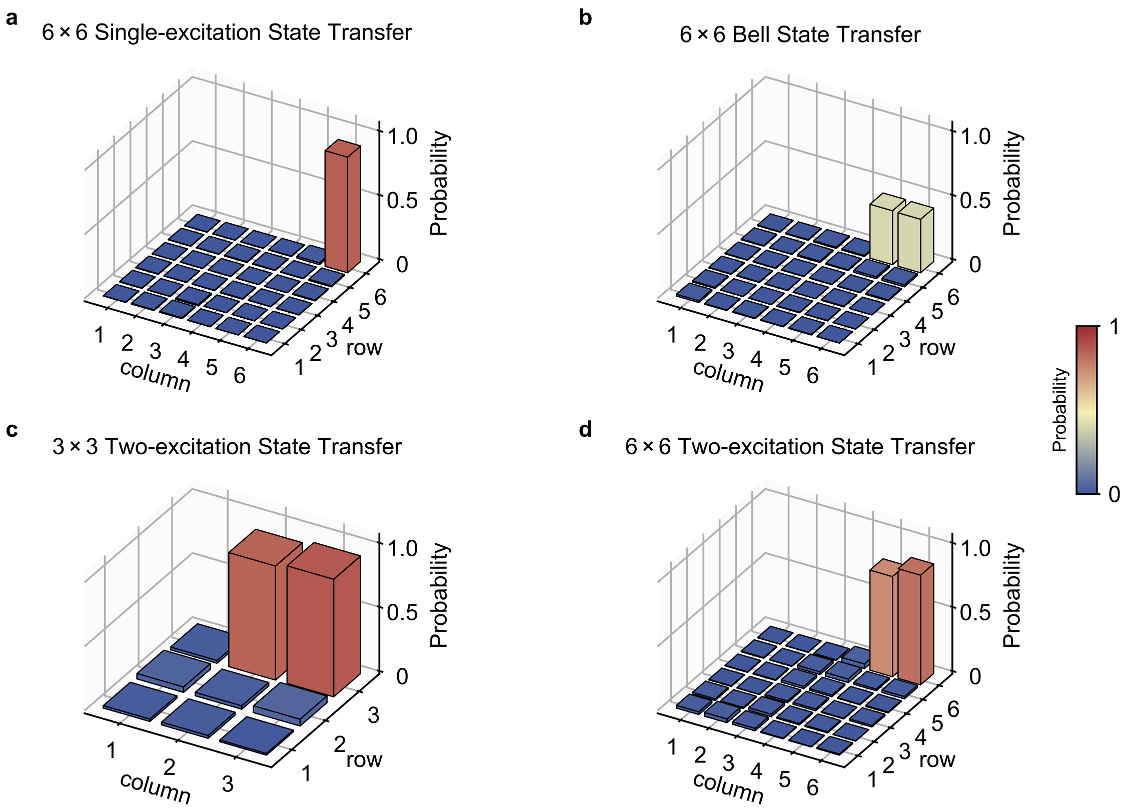

Supplementary Figure 9. **Measured qubit excited-state populations at** t_{QST}**. a-d**, Histograms of the measured excited-state populations at time t_{QST} for the 6×6 single-excitation state transfer, 6×6 Bell state transfer, 3×3 two-excitation state transfer, and 6×6 two-excitation state transfer. The height of solid bars denotes the measured probability of $|1\rangle$ state for each qubit.

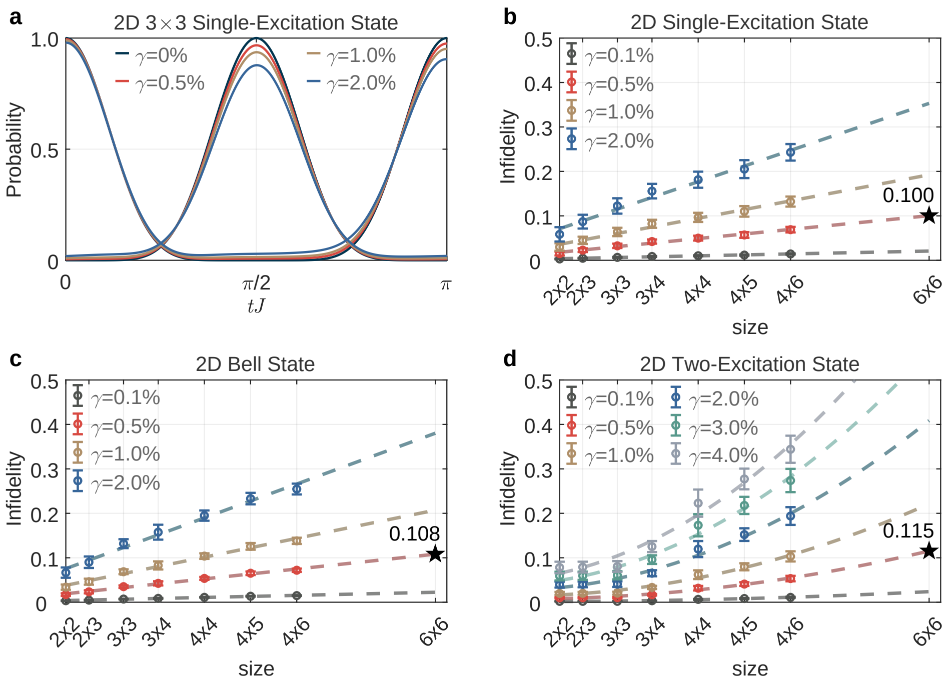

Supplementary Figure 10. **Effects of thermal populations on QST for different system sizes.** **a**, Simulated population dynamics of initial qubit Q_1 and target qubit Q_9 for the 3×3 single-excitation QST, contrasting residual thermal populations of $\gamma = 0\%, 0.5\%, 1\%$, and 2%. The result for each γ is the average over 25 random realizations. System-size dependence of infidelity computed at $tJ = \pi/2$ for transferring a single-excitation state (**b**), a Bell state (**c**), and the two-excitation state (**d**). In the numerical simulations, we use ideal 2D lattices without defects or cross-couplings. We adopt the coupling protocol of Ref. [1] for single-excitation and Bell state QST, and Monte Carlo annealing protocol for transferring two-excitation state. In panels **b**, **c**, and **d**, circle markers are the numerical results, and the dashed lines are the fitting results. Single-excitation and Bell state results are fitted with a linear function, while two-excitation results are fitted with a quadratic function. Each data point is the average over 25 random realizations, and error bars are the standard deviation. Black stars indicate the extrapolated QST infidelities in the 6×6 network with $\gamma = 0.5\%$.

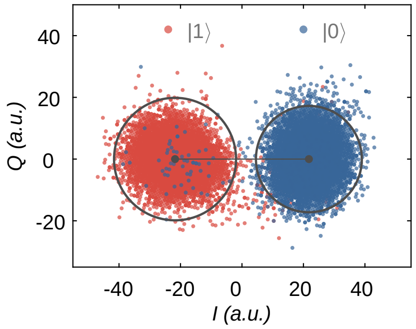

Supplementary Figure 11. **Measurements of thermal populations.** We estimate the residual thermal populations by preparing the qubit in $|0\rangle$ ($|1\rangle$) state and promptly measuring it. Repeating this process 12,000 times for $|0\rangle$ ($|1\rangle$) state, we plot the demodulated readout signals on the I-Q plane [5], where a.u. is the abbreviation of arbitrary units, which means the obtained signal amplitude is not the exact value of the real-world signal but proportional to it. Each blue (red) point represents a single measurement of $|0\rangle$ ($|1\rangle$) state. About 0.5% of blue points fall into the red region of $|1\rangle$ state, which approximately represents the residual thermal populations.

representations of the spin operators $\hat{S}_{1,\alpha}$ and $\hat{S}_{2,\alpha}$ ($\alpha = x, y, z$) are

$$\hat{S}_{1,x} = \frac{1}{\sqrt{2}}\begin{pmatrix} 0 & 1 & 0 \\ 1 & 0 & 1 \\ 0 & 1 & 0 \end{pmatrix} \otimes \begin{pmatrix} 1 & 0 & 0 \\ 0 & 1 & 0 \\ 0 & 0 & 1 \end{pmatrix}, \quad \hat{S}_{1,y} = \frac{1}{\sqrt{2}}\begin{pmatrix} 0 & -i & 0 \\ i & 0 & -i \\ 0 & i & 0 \end{pmatrix} \otimes \begin{pmatrix} 1 & 0 & 0 \\ 0 & 1 & 0 \\ 0 & 0 & 1 \end{pmatrix}, \quad \hat{S}_{1,z} = \begin{pmatrix} 1 & 0 & 0 \\ 0 & 0 & 0 \\ 0 & 0 & -1 \end{pmatrix} \otimes \begin{pmatrix} 1 & 0 & 0 \\ 0 & 1 & 0 \\ 0 & 0 & 1 \end{pmatrix}, \tag{1}$$

$$\hat{S}_{2,x} = \begin{pmatrix} 1 & 0 & 0 \\ 0 & 1 & 0 \\ 0 & 0 & 1 \end{pmatrix} \otimes \frac{1}{\sqrt{2}}\begin{pmatrix} 0 & 1 & 0 \\ 1 & 0 & 1 \\ 0 & 1 & 0 \end{pmatrix}, \quad \hat{S}_{2,y} = \begin{pmatrix} 1 & 0 & 0 \\ 0 & 1 & 0 \\ 0 & 0 & 1 \end{pmatrix} \otimes \frac{1}{\sqrt{2}}\begin{pmatrix} 0 & -i & 0 \\ i & 0 & -i \\ 0 & i & 0 \end{pmatrix}, \quad \hat{S}_{2,z} = \begin{pmatrix} 1 & 0 & 0 \\ 0 & 1 & 0 \\ 0 & 0 & 1 \end{pmatrix} \otimes \begin{pmatrix} 1 & 0 & 0 \\ 0 & 0 & 0 \\ 0 & 0 & -1 \end{pmatrix}. \tag{2}$$

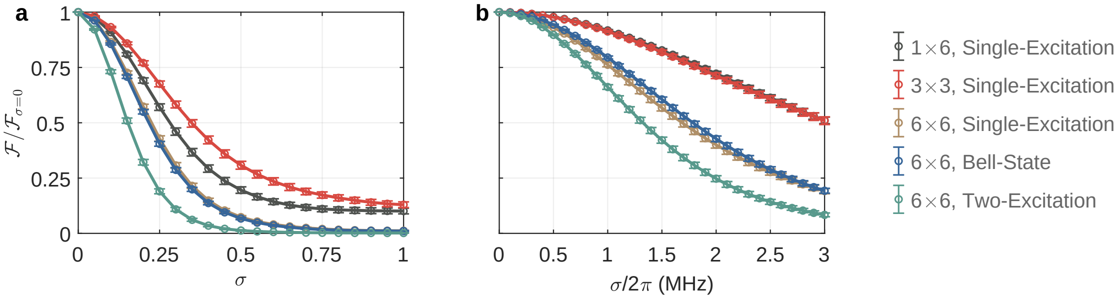

Supplementary Figure 12. **Noise analysis for QST. a**, Relative QST fidelity compared to the clean case after adding $\{\delta_{m,n}J_{m,n}\}$ to the NN couplings $\{J_{m,n}\}$ for different cases studied in the main text, where $\delta_{m,n}$ is sampled from a Gaussian distribution $N(0,\sigma)$. 6×6 two-excitation case shows the most sensitive trend under the same coupling noise deviation σ compared with other cases. **b**, Relative QST fidelity compared to the clean case after adding Gaussian noise $\delta\sim N(0,\sigma)$ to the qubit frequencies. A similar trend is observed, where the 6×6 two-excitation displays the largest decay in comparison to the ideal scenario. Each data point is averaged over 200 random instances, and error bars represent the standard error of the statistical mean.

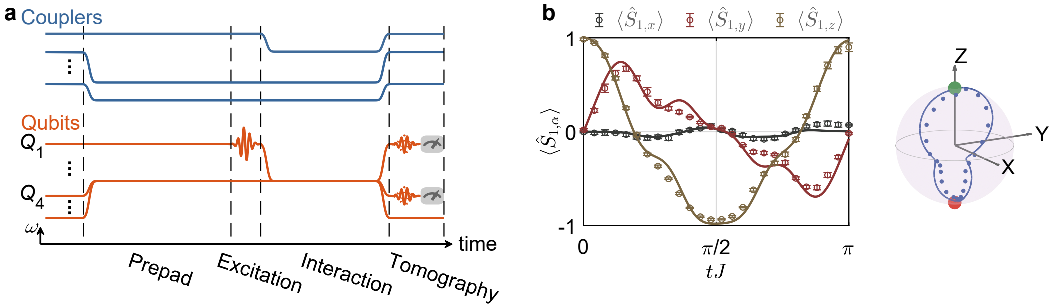

Supplementary Figure 13. **Trajectories of QST in large-spin representation. a**, Cartoon schematic of typical pulse sequences for two-qubit tomographic measurements of QST dynamics. Note that we take Q_1 and Q_4 as an example here. The ω axis indicates the frequency domain of couplers (blue curves) and qubits (orange curves). The time axis includes four steps. First, we apply square pulses on all other qubits except Q_1 and all couplers non-neighbor to Q_1 to bring them into QST work point. To suppress the small pulse distortion caused by step responses, which may disturb the QST process, we wait for 2 μs (prepad length) before exciting Q_1 with a π pulse (orange Gaussian-type waveform) and tuning it to interaction frequency ω_I. After a QST with a time of t, we apply microwave pulses, selected from $\{I, X/2, Y/2\}^{\otimes 2}$ to target qubits, Q_1 and Q_4, for tomographic measurements. **b**, Measured trajectories for $\hat{S}_1$ in the 3×3 network with the QST-optimized couplings (see Fig. 2**g** of the main text). Left panel shows the dynamics of $\langle\hat{S}_{1,x}\rangle$, $\langle\hat{S}_{1,y}\rangle$, and $\langle\hat{S}_{1,z}\rangle$. The right panel shows the corresponding trajectory on the Bloch sphere. Circles and solid lines represent experimental and numerical results, respectively. Error bars are the standard deviation of five experimental repetitions.

A quantum state $|\psi\rangle$ of the two spins has the following expectation values

$$\langle\hat{S}_{1,x}\rangle\,,\ \langle\hat{S}_{1,y}\rangle\,,\ \langle\hat{S}_{1,z}\rangle\,,\ \langle\hat{S}_{2,x}\rangle\,,\ \langle\hat{S}_{2,y}\rangle\,,\ \langle\hat{S}_{2,z}\rangle\,, \tag{3}$$

where the expectation value $\langle\hat{O}\rangle$ of an operator $\hat{O}$ is given by $\langle O\rangle = \langle\psi|\hat{O}|\psi\rangle$. In the single-excitation-conserved subspace of 9 qubits, $|\psi(t)\rangle = \sum_{i=1}^{9} a_i(t)|\phi_i\rangle$, where $|\phi_i\rangle = (0,\ldots,1,\ldots,0)^{\mathrm{T}}$ is a basis vector of the subspace, such that the i-th qubit is excited while the remaining ones are in their ground states. We can thus conveniently represent expectation values of the spin operators using the coefficients of $|\psi\rangle$. Taking $\hat{S}_{1,x}$ for example, we have

$$\begin{aligned}\langle\hat{S}_{1,x}\rangle =&(a_1a_4{}^* + a_4a_1{}^* + a_2a_5{}^* + a_5a_2{}^* \\ &+ a_3a_6{}^* + a_6a_3{}^* + a_4a_7{}^* + a_7a_4{}^* \\ &+ a_5a_8{}^* + a_8a_5{}^* + a_6a_9{}^* + a_9a_6{}^*)/\sqrt{2}.\end{aligned} \tag{4}$$

Each term $a_ia_j{}^*$ corresponds to an element of a two-qubit density matrix $\rho_{(Q_i,Q_j)}$, which can be easily extracted using tomographic measurements.

Supplementary Figure 13**a** shows an example of experimental sequences for the tomographic measurements of the qubit pair (Q_1, Q_4). The measurements for other qubit pairs are similarly obtained by applying the tomographic pulses to the corresponding qubits. Supplementary Figure 13**b** presents the experimentally measured dynamics of $\langle\hat{S}_{1,x}\rangle$, $\langle\hat{S}_{1,y}\rangle$, $\langle\hat{S}_{1,z}\rangle$ and the trajectories on the Bloch sphere for the QST in 3×3 network with the optimized coupling configurations (see Figs. 3**h** and **i** of the main text), which are in good agreement with the numerics.

SUPPLEMENTARY NOTE 6 - PREPARATION OF BELL STATE

The initial Bell state in Fig. 3**b** of the main text is prepared by applying a quantum circuit to Q_1 and Q_2, which is shown in Supplementary Figure 14**a**. Entanglement is generated by a two-qubit controlled-Z (CZ) gate, which is implemented by dynamically tuning on/off the interaction between qubit energy levels of $|11\rangle$ and $|20\rangle$. The corresponding pulse sequences are shown in Supplementary Figure 14**b**. A good preparation of a Bell state largely relies on the quality of CZ and single-qubit gates. We use the cross-entropy benchmarking (XEB) technique [7] to characterize the gate errors and the results are shown in Supplementary Figure 14**c**. Pauli errors of ~0.064% and ~ 0.73% are extracted for single-qubit gates and CZ gate, respectively. The prepared initial Bell state is further confirmed by the reconstructed density matrix using quantum state tomography (see Fig. 3**b** of the main text), which yields a fidelity of ~0.992.

Due to the finite step edge times of square pulses for realizing QST, the Bell state can accumulate an unwanted dynamical phase during the frequency shifts of Q_1 and Q_2. To guarantee the initial Bell state for QST is $|\Psi^-\rangle = (|01\rangle - |10\rangle)/\sqrt{2}$ at the beginning of the interaction, the first $\pi/2$ rotation around y axis of Q_1 is replaced by a $\pi/2$ rotation around the axis with an additional phase ϕ relative to the y-axis.

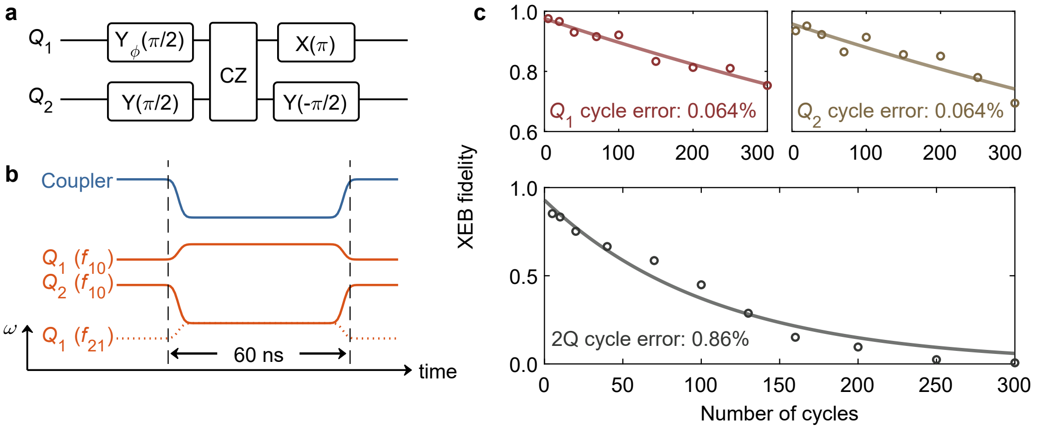

Supplementary Figure 14. **Preparation of the Bell state with CZ gate.** **a**, Quantum circuits for preparing a Bell state between Q_1 and Q_2. $Y(\theta)$ ($X(\theta)$) denotes rotating the state an angle θ around the y-axis (x-axis) of the Bloch sphere. $Y_\phi(\theta)$ rotates the state by an angle θ around the axis which has a phase ϕ relative to the y-axis of the Bloch sphere. **b**, Pulse sequences for implementing a two-qubit CZ gate assisted with a tunable coupler. The gate time is about 60 ns in the experiment. **c**, Cross-entropy benchmarking of single-qubit gates and two-qubit CZ gate. Pauli errors of single-qubit gates for Q_1 and Q_2 are benchmarked by simultaneous XEB. For the CZ gate, each cycle contains two parallel single-qubit gates and a subsequent CZ gate. Pauli error of CZ gate extracted here is ~ 0.73%.

SUPPLEMENTARY NOTE 7 - (NON)ERGODICITY OF TWO-EXCITATION HAMILTONIANS

An often-used analysis to classify quantum chaotic properties of Hamiltonians of interest is to investigate the degree of level repulsion of their eigenvalues $\{E_\alpha\}$. While typical ergodic Hamiltonians exhibit a Wigner-Dyson-like distribution $P(s)$ for the gaps between nearest eigenvalues ($s_\alpha \equiv E_{\alpha+1} - E_\alpha$), non-ergodic ones show, on the other hand, absence of level repulsion; the statistical properties encoded in $P(s)$ follow a Poisson distribution instead [8]. To avoid the complication of unfolding the spectrum to guarantee a unit mean-level spacing, we take instead the ratio of adjacent gaps $r_\alpha \equiv \min(s_\alpha, s_{\alpha+1})/\max(s_\alpha, s_{\alpha+1})$ [9]. In this case, the ergodic and non-ergodic (Poisson) distributions translate, respectively, to [10]

$$P_{\rm GOE}(r) = \frac{27}{8}\frac{r + r^2}{(1 + r + r^2)^{5/2}}\ , \quad P_{\rm P}(r) = \frac{2}{1 + r^2}\ , \tag{5}$$

where we focus on the case of the class of random matrices belonging to the Gaussian orthogonal ensemble (GOE), which are real and symmetric.

A complication in this exploration arises when existing symmetries of the Hamiltonian spoil the classification of its ergodic properties if they are not resolved [11]. In particular, as explained in the main text, we enforce a real-space inversion-symmetry constraint for the Hamiltonians we engineer, which, in practice, makes its spectrum independently subdivided into sectors whose parity is even or odd under this point group symmetry. By independently constructing the Hamiltonian in each of these subsectors, we contrast the gap distributions for the cases where the couplings are either randomly selected within a uniform

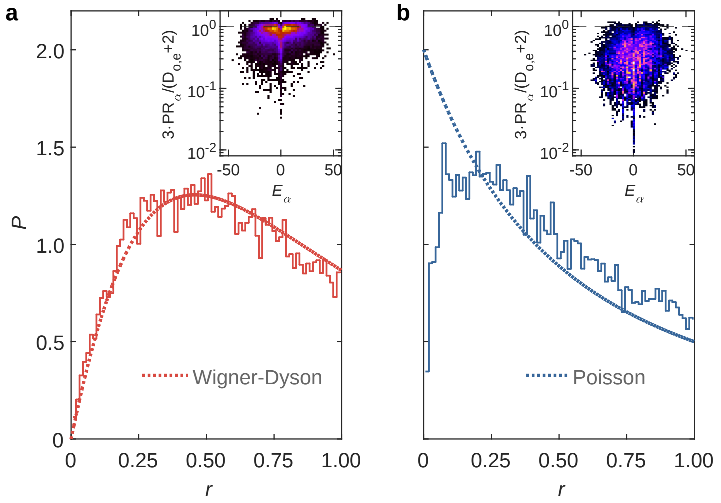

Supplementary Figure 15. **Ergodicity analysis of random and optimized couplings for QST.** The distribution of the ratio of adjacent gaps r for two excitations in the 6×6 qubit network: **a**, for the case of random couplings, and **b**, for the solutions extracted from the optimization process that maximizes the quantum state transfer at later times. Owing to the inversion symmetry of the couplings (nearest-neighbors or across plaquettes), even (e) and odd (o) sectors are resolved. Dashed lines give the Wigner surmise for $P(r)$ and the Poisson distribution, respectively. Forty realizations of both random couplings and optimized solutions are averaged. The corresponding insets give a two-dimensional histogram of the participation ratio of each eigenstate $|\alpha\rangle$ in the computational basis for the whole ensemble of solutions, normalized by the Hilbert dimensions $\mathcal{D}_{o,e}$ [$\mathcal{D}_o = 306$ and $\mathcal{D}_e = 324$]; brighter colors are associated with higher counts, and horizontal lines mark the GOE prediction extracted from the theory of random matrices. The matrices used in both cases have bounds on the nearest-neighbor couplings given by $[J_{\min}, J_{\max}] = [-10, -0.1]$ MHz.

distribution $[J_{\min}, J_{\max}]$ or when they are optimized (within the same bounds) to achieve a quantum state transfer at times $t_{\rm QST} = \pi/(2J)$; the corresponding distributions $P(r)$ are reported in Supplementary Figure 15. They show that for solutions that optimize the QST, here selected to have fidelities $\mathcal{F} > 0.93$, $P(r)$ approaches a Poisson distribution (i.e., quasi-nonergodic), unlike the cases with random couplings, more closely similar to the GOE surmise.

Additionally, we investigate ergodic properties of the eigenstates themselves (insets in Supplementary Figure 15), via the quantification of the participation ratio in the computational basis (inversion-symmetric Fock states $|n\rangle$)

$$\mathrm{PR}_\alpha = \frac{1}{\sum_{n=1}^{\mathcal{D}_{o,e}} |c_\alpha^n|^4}\,, \tag{6}$$

where $c_\alpha^n = \langle n|\alpha\rangle$. Random matrices of the GOE class exhibit $\mathrm{PR} = \frac{\mathcal{D}+2}{3}$ [12, 13]. The typical eigenstates from the Hamiltonian with optimized couplings deviate from this prediction much more markedly than do those with random couplings, as seen in the insets of Supplementary Figure 15, showing a two-dimensional histogram of all eigenstates for the forty realizations considered and noting the logarithmic scale.

This analysis makes it immediately apparent that when random couplings are used, the ensuing spectral properties exhibit characteristics of ergodic systems; the same would be the case even if homogeneous couplings in our Hamiltonian were used instead (if all point-group symmetries were resolved). Conversely, optimizing the coupling parameters to achieve a high-fidelity QST converges to coupling matrices $\{J_{m,n}\}$ whose associated Hamiltonian avoids this ergodic fate and steers it towards nonergodicity.

A physical picture is useful to understand this difference. In ergodic Hamiltonians, one expects that the evolution governed by $e^{-\mathrm{i}\hat{H}t}$ leads to a diffusive exploration of the Hilbert space with time t when the initial state is a single point in this space (Fock state). In contrast, non-ergodic Hamiltonians typically exhibit characteristic revivals throughout the dynamics, leading them to the possibility of exploring states arbitrarily close to the initial conditions, for example. Similarly, one can engineer (quasi) non-ergodic Hamiltonians such that at a certain time, the majority of the contribution to $|\psi(t)\rangle$ resides in another single Fock state (point in the Hilbert space). This is the procedure envisaged by the quantum state transfer.

To illustrate this, we show in Supplementary Figures 16**a,** and 16**b** the dynamics of an initially prepared two-excitation state with optimized and random couplings, respectively. This is schematically seen as the weight of $|\psi(t)\rangle$ in each Fock state $|n\rangle$ for various times. This expands the original description in the main text, but now for a 6×6 qubit network, and its corresponding $\mathcal{D}_{\hat{H}} = \binom{36}{2} = 630$ Fock states. While the QST is seen in the case of optimized couplings, manifested as a revival of the maximum weight in a Fock state at times $tJ = \pi/2$, random couplings fail to have an instant of time where the projection is accumulated in any single $|n\rangle$ at $t > 0$. It indicates that the dynamics occur diffusively over the Fock space if random couplings are taken.

To explore this analogy further, we use a metric for distances between Fock states, introduced in the Methods Section in the main text. For completeness, we partially repeat this description here. The idea is to establish an associated time for a Fock state to be reached, given the typical coupling's matrix values – a distant Fock state would thus take numerous hopping times to be accessed. If using the initial state as a reference, we defined a distance as $d(|0\rangle, |n'\rangle) = \frac{1}{4}\sum_{l=1}^{2}\left(|x'_l - x_0| + |y'_l - y_0| + |x'_l - x_1| + |y'_l - y_1|\right) - 1/2$, where (x'_l, y'_l) are the cartesian coordinates of each of the l-excitations ($l = 2$) of a generic Fock state $|n'\rangle$. For the initial state $|n = 0\rangle$, one thus have (x_0, y_0) and (x_1, y_1) being the coordinates of its excitations. Hence $d(|0\rangle, |0\rangle) = 0$ whereas $d(|0\rangle, |n_{\rm target}\rangle) = 8.5$ for the target state in this network size.

An average distance can be dynamically defined as

$$\langle d(t)\rangle = \sum_{n=0}^{630-1} d(|0\rangle, |n\rangle)\, |\langle n|\psi(t)\rangle|^2 \; ; \tag{7}$$

likewise, the root-mean-square σ of the wave-packet spreading in the Fock basis is

$$\langle \sigma(t)\rangle = \sqrt{\sum_{n=0}^{630-1} d^2(|0\rangle, |n\rangle)\, |\langle n|\psi(t)\rangle|^2} \; . \tag{8}$$

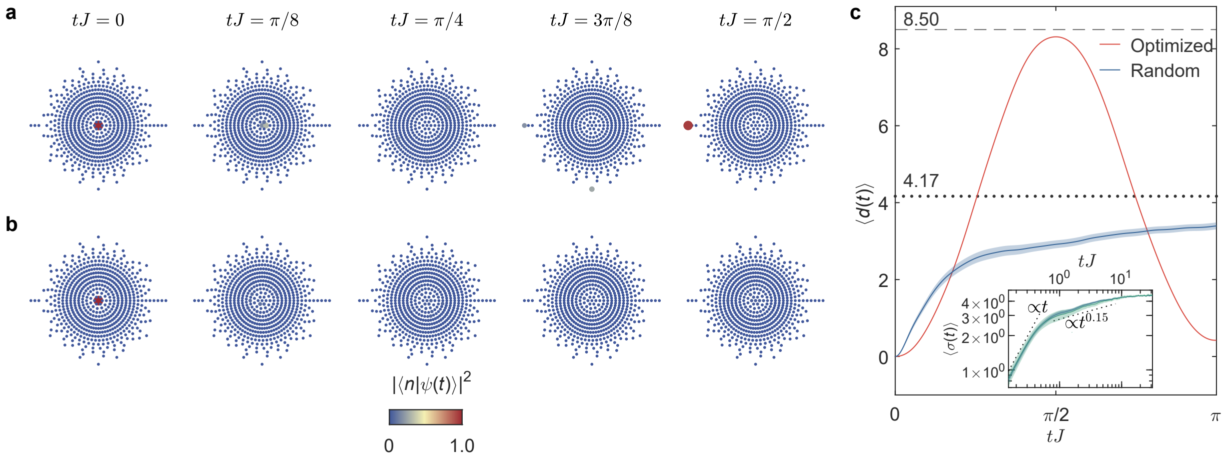

Supplementary Figure 16. **Dynamics of wave-packets in Fock space. a,** Projection of the time-evolved wavefunction in Fock space, $|\langle n|\psi(t)\rangle|^2$ for the case where the couplings $\{J_{m,n}\}$ were optimized to maximize the quantum state transfer at times $tJ = \pi/2$ [$J = 1$ MHz here], taking into account both the cross-couplings $J^\times$ and the defective coupler; different time-snapshots are marked. Here, the radial direction is given by $d(|0\rangle, |n\rangle)$ (see text) while the angular one is arbitrary; $|\langle n|\psi(t)\rangle|^2$ is mapped by the color bar and the size of the marker at each Fock state $|n\rangle$. **b,** The same for the case of random couplings: Here, the wave-packet diffuses in Fock space. **c,** The dynamics of the wave-packet's 'center of mass' for optimized and random couplings; the inset gives the wave-packet spread in time for the case of the random couplings, contrasting 6×6 (blue) and 12×12 (green), (averaged over 40 and 10 realizations, respectively), with regimes marking an initial ballistic transient and another of subdiffusive spreading. The horizontal dashed (dotted) line in the main panel depicts the $d(|0\rangle, |n_{\rm target}\rangle)$ $(\overline{d})$ value – see text. The matrices used in both cases have bounds on the nearest-neighbor couplings given by $[J_{\rm min}, J_{\rm max}] = [-10, -0.1]$ MHz.

Supplementary Figure 16**c** displays the time-evolution of both quantities, contrasting the case of an optimized solution of the couplings with the averaged value over 40 realizations of random couplings instances. The average distance $\langle d(t)\rangle$ measures the evolution of the wave-packet 'center of mass' in Fock space: For QST-optimized couplings, one observes a ballistic (almost) periodic evolution between the initial state $|0\rangle$ and the target state $|n_{\rm target}\rangle$. Conversely, a slow evolution towards the mean distance $\overline{d} = \frac{1}{630}\sum_{n=0}^{630-1} d(|0\rangle, |n\rangle)$ is seen in the case of randomly chosen $\{J_{m,n}\}$. These results are similar to the theory/experimental ones described in Fig. 4 in the main text for a smaller qubit network size, 3×3.

A refined characterization of the propagation in Fock space is given by σ, which typically exhibits a time-dependence $\propto t^\alpha$. For $\alpha = 1/2 (\alpha < 1/2)$, the wave-packet propagates diffusively (sub-diffusively) in Fock space, while $\alpha = 1$ describes ballistic transport. We note that at short-time scales (inset in Supplementary Figure 16**c**), a transient ballistic propagation is observed that gives way to a subdiffusive one at later times before the effects of the subspace's finiteness set in at $tJ \gg 1$. Such a mix of ballistic and diffusive behavior is similarly observed in (excitation) number-conserving random unitary circuits [14], with random gates. These are not equivalent to the unitary evolution with random couplings that we perform here, that is, with a fixed functional form of the gates but random amplitudes. However, they do share similarities (at least in short times) if one performs a Trotterization of the dynamical evolution. Whether the subdiffusive spread in intermediate time scales we observe (as opposed

to diffusive) is affected by the system's finiteness or the small number of excitations we consider deserves future investigation. Likewise, random amplitude unitary circuits being dissimilar from random unitary circuits, the corresponding expected operator spreading, as investigated in Ref. [14], is hitherto unknown.

SUPPLEMENTARY NOTE 8 - NUMERICALLY OPTIMIZED QUANTUM STATE TRANSFER WITH MONTE CARLO ANNEALING

In the main text, to realize a high-fidelity QST in a large, imperfect two-dimensional quantum network where unwanted cross-couplings and a defective coupler exist, we employ a Monte Carlo annealing procedure to optimize NN couplings of the network. With the numerically optimized result as a reference, we experimentally calibrate our superconducting quantum processor to demonstrate the quantum state transfer of few excitations and the physical insight behind it. In this section, we provide the optimized coupling values and the corresponding QST dynamics for the cases of single-excitation (see Supplementary Figure 17 and Supplementary Figure 18), Bell state (see Supplementary Figure 19), and two excitations (see Supplementary Figures 20 and 21). In two of these solutions, we release the inversion symmetry constraint to achieve a slightly higher QST-fidelity, given that more degrees of freedom is beneficial for the optimization procedure.

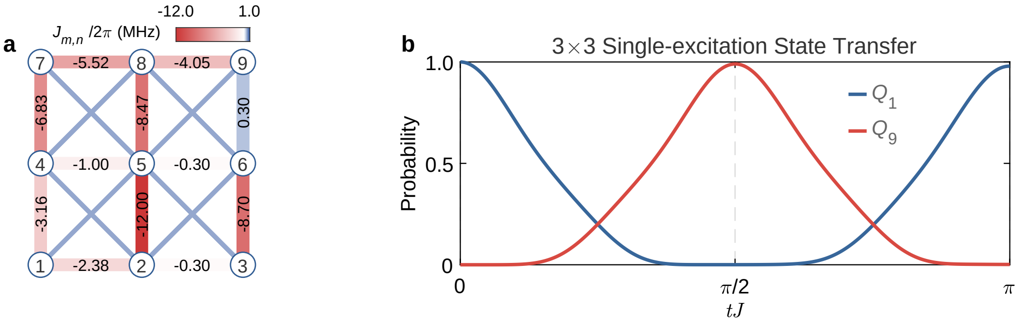

Supplementary Figure 17. **Numerically optimized couplings for single-excitation QST in the** 3×3 **network. a**, Numerically optimized NN couplings. **b**, The corresponding dynamics for transferring single excitation from Q_1 to Q_9, with a maximum QST fidelity of ~ 0.9902.

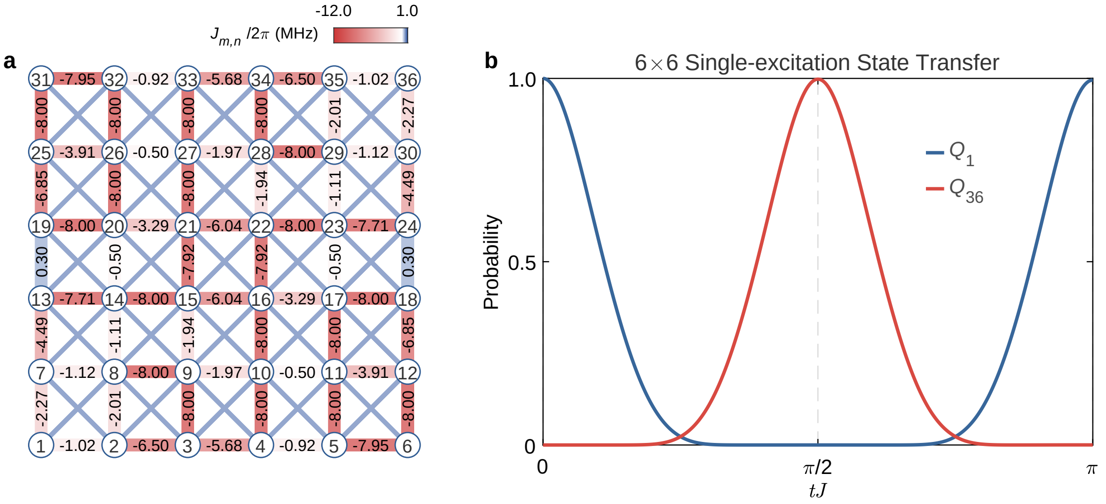

Supplementary Figure 18. **Numerically optimized couplings for single-excitation QST in the** 6×6 **network. a**, Numerically optimized NN couplings. **b**, The corresponding dynamics for transferring single excitation from Q_1 to Q_{36}, with a maximum QST fidelity of ~ 0.9979.

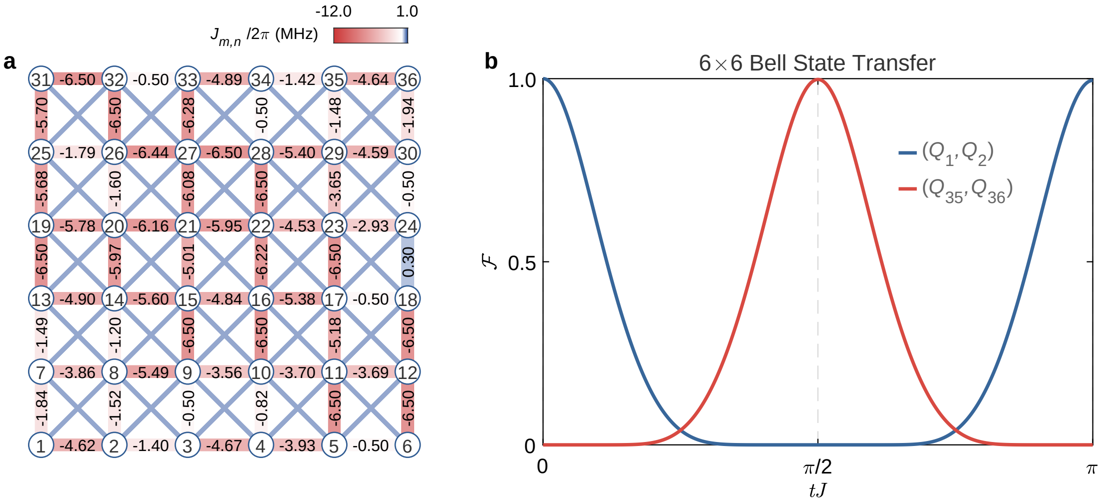

Supplementary Figure 19. **Numerically optimized couplings for Bell state QST in the** 6×6 **network.** **a**, Numerically optimized NN couplings. **b**, The corresponding dynamics for transferring Bell state from (Q_1, Q_2) to (Q_{35}, Q_{36}), with a maximum QST fidelity of ~ 0.9978.

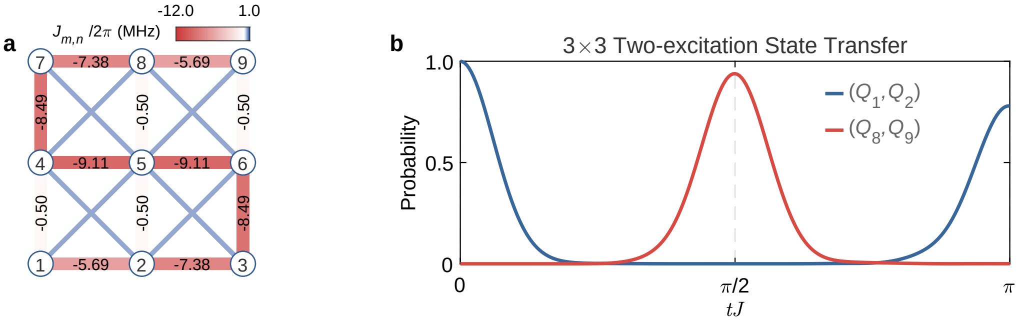

Supplementary Figure 20. **Numerically optimized couplings for two-excitation QST in the** 3×3 **network.** **a**, Numerically optimized NN couplings. **b**, The corresponding dynamics for transferring two excitations from (Q_1, Q_2) to (Q_8, Q_9), with a maximum QST fidelity of $\sim$ 0.9388.

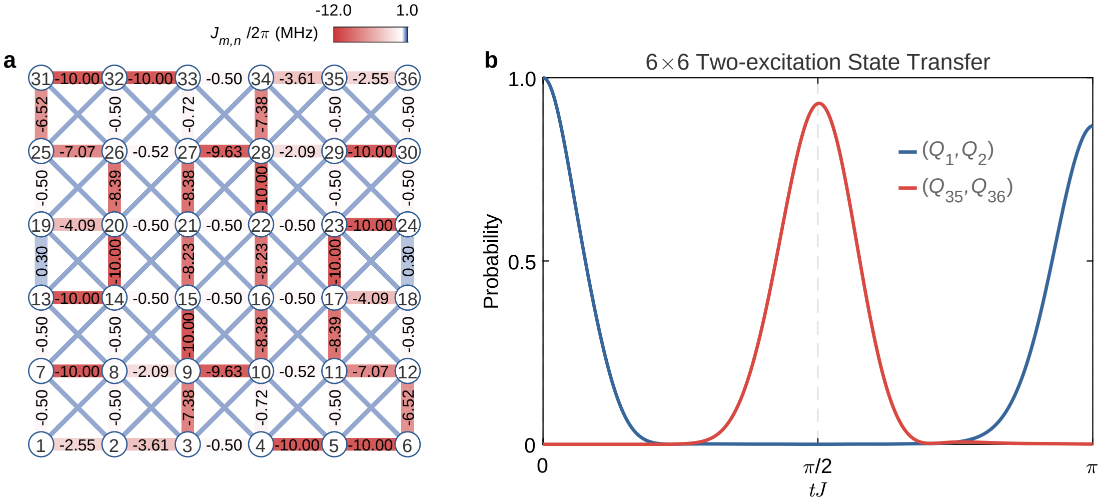

Supplementary Figure 21. **Numerically optimized couplings for two-excitation QST in the** 6×6 **network.** **a**, Numerically optimized NN couplings. **b** The corresponding dynamics for transferring two excitations from (Q_1, Q_2) to (Q_{35}, Q_{36}), with a maximum QST fidelity of $\sim$ 0.9301.

SUPPLEMENTARY NOTE 9 - QUANTUM SPEED LIMIT BOUNDS

In the main text, we argue that the quantum state transfer cannot occur arbitrarily fast but rather needs to, at least, take place at times equal to the minimum orthogonalization time, whose values are shown in the Figs. 2, 3, and 4 of the main text. Such an orthogonalization time may depend on either the (time-conserved) mean energy E (with respect to the ground-state energy) or the energy uncertainty ΔE. In addition, the actual overlap of the time-evolving wave function with the initial state is bounded, as demonstrated by Margolus and Levitin (ML) [15] for the case of the mean energy-bound,

$$|\langle\psi(0)|\psi(t)\rangle| \geq \cos\left(\sqrt{\frac{\pi E t}{2\hbar}}\right), \tag{9}$$

or in the ΔE-bounded dynamics, as shown by Mandelstam and Tamm (MT) [16]

$$|\langle\psi(0)|\psi(t)\rangle| \geq \cos\left(\frac{\Delta E t}{\hbar}\right). \tag{10}$$

To explore this further, we report in Supplementary Figure 22 the dynamics of the four cases studied to accomplish QST. In all situations, the MT-bound governs the dynamics, and the tighter bound is in the case of the two-excitation state. This is seen for various solutions of the optimized couplings that maximize the QST fidelity. Nevertheless, it is important to emphasize that generically, it is always possible to make the dynamics governed by the ML bound, provided that the initial state has associated mean energy sufficiently close to the ground state of the emulated Hamiltonian. For the initial states we considered here, that was never the case.

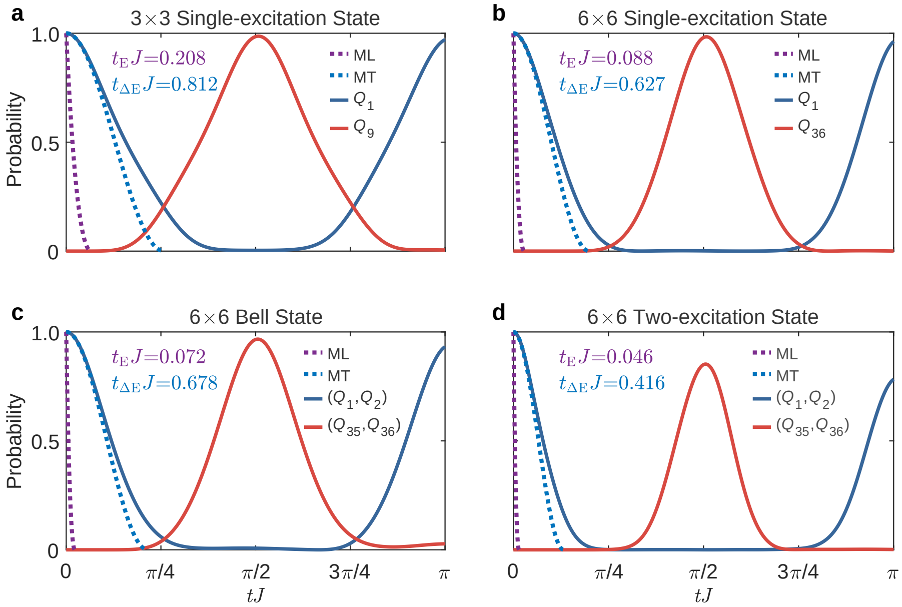

Supplementary Figure 22. **Quantum speed limit bounds for 2D optimized couplings. a-d**, Analysis of the quantum speed limits bounds [Supplementary Equation (9) and (10)] for the QST optimized couplings of **a,** 2D 3×3 single-excitation state, **b,** 2D 6×6 single-excitation state, **c,** 2D 6×6 Bell state and **d,** 2D 6×6 two-excitation state. The actual population dynamics for the initial and final qubits are also included. The former is limited from below by the MT bound. The values of $t_{\Delta E}$ and t_E annotated in each panel stand for the minimal time given by the quantum speed limit of the corresponding MT and ML bounds, respectively.

SUPPLEMENTARY NOTE 10 - OPTIMIZATION SOLUTIONS FOR MUCH LARGER SYSTEM SIZES

As described in the main text, we employ a Monte Carlo annealing optimization process on the space of parameters of the tunable couplings to guide the experiments in accomplishing an efficient QST. This optimization is performed via minimizing a cost function, the infidelity of the transfer at $t_{\rm QST}$ to a target state $|\psi_{\rm target}\rangle$, $\tilde{F}(t_{\rm QST}) = 1 - |\langle\psi(t_{\rm QST})|\psi_{\rm target}\rangle|^2$. There are two main constraints to the success of this approach. The first is numerical, in which one needs an efficient computation of the unitary $e^{-{\rm i}\hat{H}t_{\rm QST}}$ operator necessary to obtain $|\psi(t_{\rm QST})\rangle = e^{-{\rm i}\hat{H}t_{\rm QST}}|\psi(0)\rangle$. In principle, one can directly do so via its eigendecomposition,

$\hat{U}e^{-i\hat{D}t_{\rm QST}}\hat{U}^\dagger$, where $\hat{D}$ is a diagonal matrix containing the eigenvalues of $\hat{H}$ (which depends on the current values of the coupling matrix) and $\hat{U}$ is the corresponding eigenvectors' matrix. While this can be obtained at a polynomial cost $[O(\mathcal{D}^3_{\hat{H}})]$ on the Hilbert space dimension $\mathcal{D}_{\hat{H}} = \binom{N}{N_{\rm exc}}$, the fact that one needs to repeatedly compute it for each proposed new coupling over the Monte Carlo sampling process, makes it a daunting task if one needs to scale it to a larger size (for the Hilbert space dimensions tackled experimentally, we emphasize this is not substantial, though). Nonetheless, tackling scalability is important, and we recall that efficient procedures exist for the computation of the action of the unitary on a state vector, particularly for the case that the matrix $\hat{H}$ is sparse, without ever having to compute the unitary itself explicitly. These are based on applying the Krylov method, which recursively creates a subspace of orthogonal vectors corresponding to the subsequent application of $\hat{H}$ in a state in this space. In the case of the exponential function, its Taylor expansion can be shown to amount to one element of the generated Krylov subspace, and thus the action of $e^{-i\hat{H}t_{\rm QST}}|\psi(0)\rangle$ can be computed efficiently. In our case, we implement this method utilizing the PETSc [17] and SLEPc [18] numerical libraries. With that, Supplementary Figure 23 and Supplementary Figure 24 shows that one can obtain efficient quantum state transfer for system sizes ranging from $N = 64$ to 1024 for single-excitations utilizing this approach, with sampling characteristics comparable to the ones utilizing the standard eigendecomposition in smaller lattice sizes ($N = 36$). Furthermore, the same can be accomplished in the two-excitation case for a 8×8 qubit network (see Supplementary Figure 25), with a Hilbert space dimension $\mathcal{D}_{\hat{H}} = \binom{64}{2} = 2016$.

The second factor that determines the success of the optimization approach refers to its efficiency: In various contexts, finding the minima of multivariate functions (the infidelity in our case) can be hampered by either the multitude of local minima that can significantly impact the search of lower values of $\tilde{F}$, trapping the sampling process in them or that, even worse, there is no significant downward slope in the optimization such that it is trapped in dubbed 'barren plateaus' [19]. While proof that barren plateaus do not impact this particular problem is elusive, our numerical solutions with different degrees of finite fidelities point towards the absence of such featureless landscapes for the system sizes and number of excitations studied.

SUPPLEMENTARY NOTE 11 - COMPARISON WITH SEQUENTIAL GATE SCHEME

Applying SWAP gates sequentially is an alternative and common scheme to transfer quantum states. To compare the QST efficiency between our work and the SWAP scheme in the settings of real devices, we perform numerical simulations for the QST realized with digital quantum gates. In practice, high fidelity iSWAP-like [7], controlled-Z (CZ) [20], and echoed cross-resonance (ECR) [21] gates have been demonstrated on multi-qubit superconducting processors. Therefore, we use parameters in these papers, including gate errors, gate length, and coherence times, to perform numerical simulations. The results for different cases are summarized in Supplementary Table 1, and the quantum circuits for single-excitation QST in a 1×6 chain are plotted in Supplementary Figure 26. Two pieces of information can be obtained from the simulation results.

- Fidelity. For QST in the 1D chain, whose Hamiltonian is integrable, our work has the best fidelity among all the cases (Supplementary Table 1), which indicates that a simple scale-up scheme by repeating the 1×6 structure in our paper can realize high-fidelity QST in larger systems. However, for imperfect 2D networks with cross-couplings and defects, the efficiency depends on gate type and gate fidelity. The transfer with iSWAP-like gate [7] yields the best fidelity because it possesses both the high gate fidelity and the short circuit depth. In contrast, the case with ECR gates [21] only apparently outperforms our scheme for two-excitation QST, where the system is non-integrable.

- Transfer time. The transfer time using digital gates largely depends on gate type and gate length. The iSWAP gate in Ref. [7] has the shortest transfer time among all cases, since it has the shortest gate length and could realize a swap operation with only one two-qubit gate. On the contrary, other cases (CZ and ECR) are far slower than our scheme. Considering a more fair comparison, where the maximum coupling of the system is $J_{\rm max}$, a single-excitation QST in an N-qubit 1D chain with consecutive NN swap operations needs a transfer time $t = \frac{\pi(N-1)}{2J_{\rm max}}$. For the pre-engineered couplings, $J_{n,n+1} = J\sqrt{n(N-n)}$, $J_{\rm max}$ appears at the middle of the chain, $J_{\rm max} = JN/2$ (assume N is an even number), whose transfer time is $t = \frac{\pi N}{4J_{\rm max}}$, about two times faster than the digital SWAP scheme.

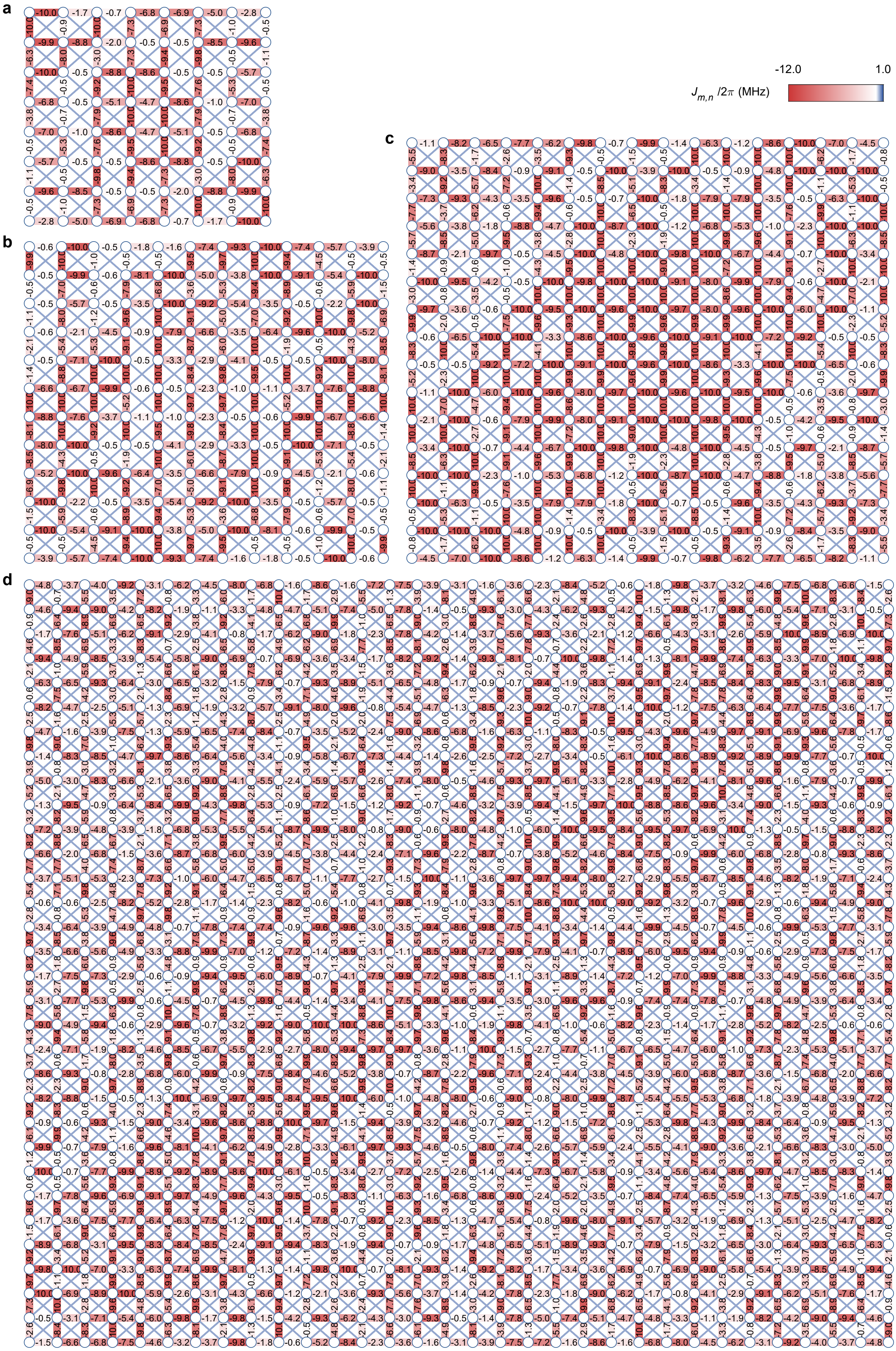

Supplementary Figure 23. **Numerically optimized couplings for one-excitation QST in large networks.** Optimized NN couplings for **a**, 8 × 8, **b**, 12 × 12, **c**, 16 × 16, and **d**, 32 × 32. The corresponding dynamics of the probabilities of initial and target states are given in Supplementary Figure 24.

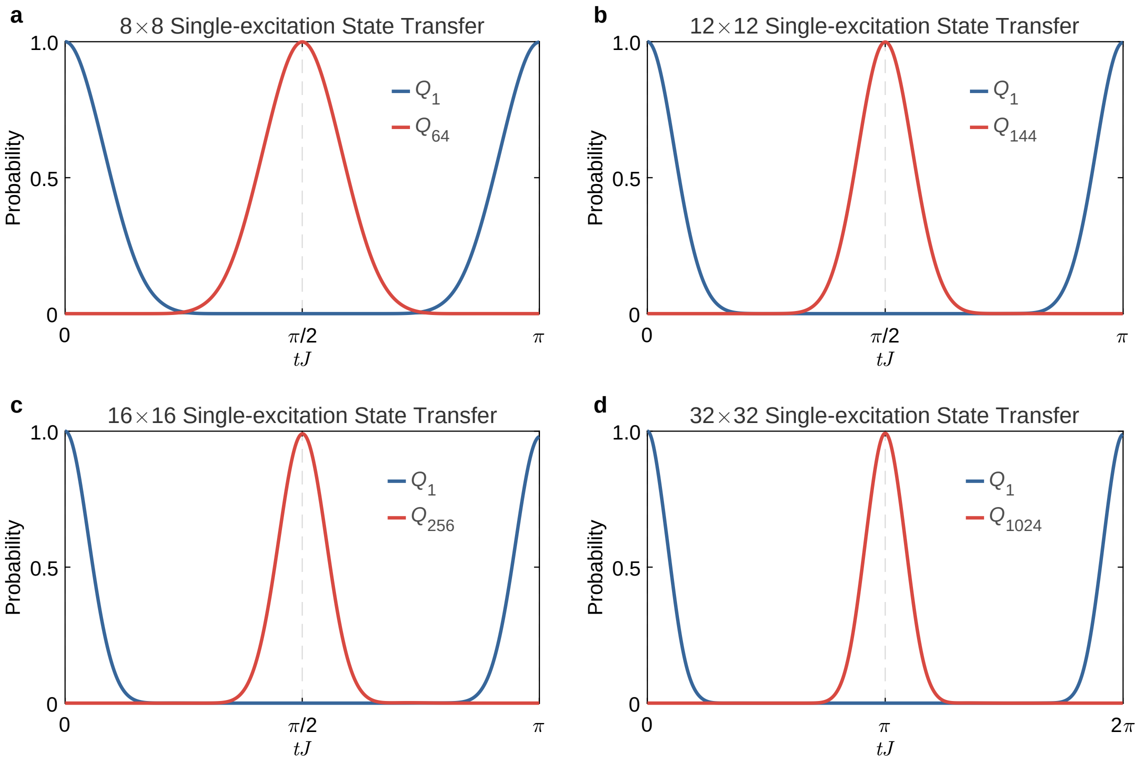

Supplementary Figure 24. **Dynamics for transferring a single excitation in large networks. a - d**, Time dynamics for transferring a single excitation from Q_1 to $Q_{N_x \times N_y}$, where $N_x = N_y = 8, 12, 16, 32$. Maximum QST fidelities are, respectively, ∼ 0.9992, ∼ 0.9987, ∼ 0.9903 and ∼ 0.9921. For the largest system size, the QST optimization is set to occur at a posterior time, $t_{\rm QST}J = \pi$ instead of $\pi/2$ as for the smaller network sizes. Since the coupling's absolute magnitudes are bounded, given the experimental capabilities, one needs to increase the transfer time if the lattice is much larger. Otherwise, the annealing process is unsuccessful in obtaining a large-fidelity solution.

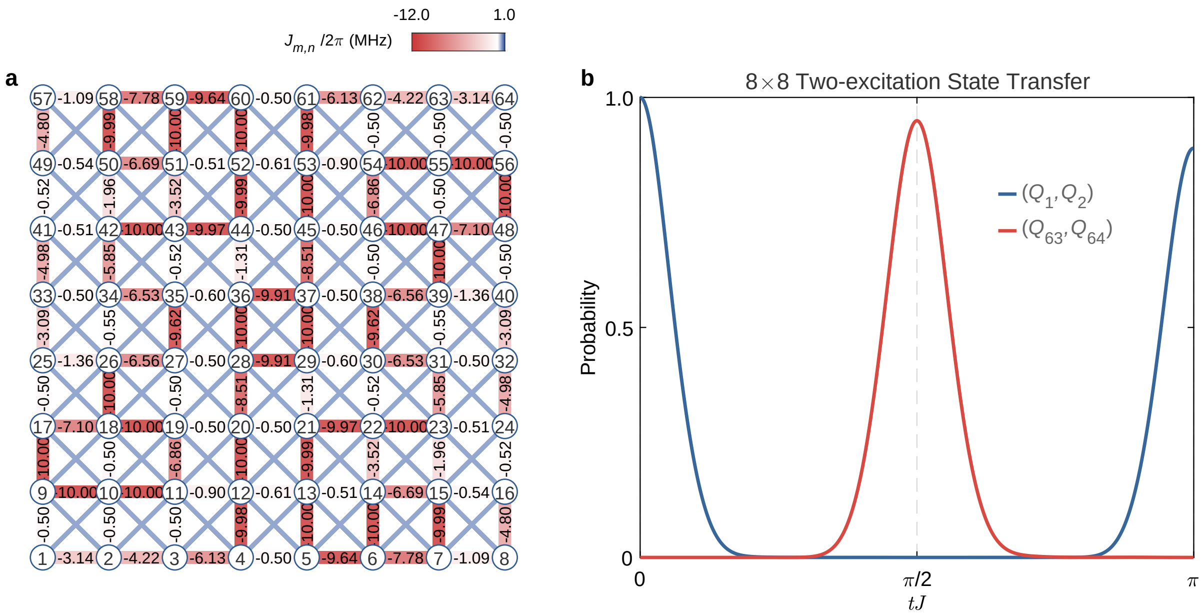

Supplementary Figure 25. **Numerically optimized couplings for two-excitation QST in the** 8×8 **network. a**, Numerically optimized NN couplings. **b**, The corresponding dynamics for transferring two excitations from (Q_1,Q_2) to (Q_{63}, Q_{64}), with a maximum QST fidelity of of ∼ 0.9493.

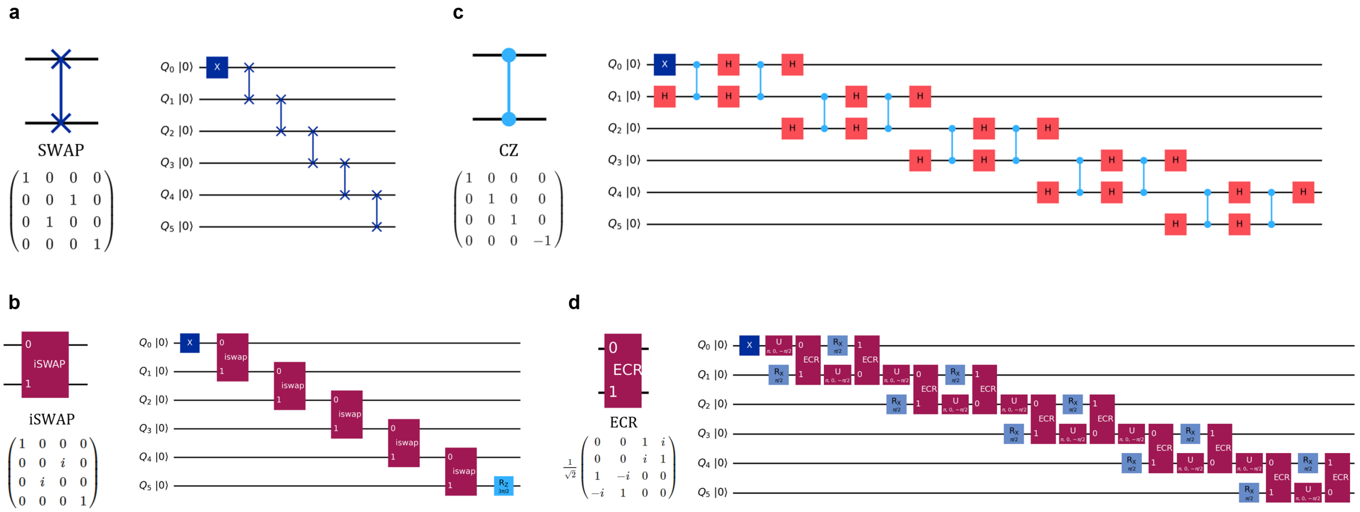

Supplementary Figure 26. **Quantum circuits for transferring a single excitation in a 1×6 chain using different types of two-qubit gates.** **a**, Five consecutive SWAP gates are applied for transferring an excitation from Q_0 to Q_5. **b-d**, By compiling the SWAP gates into other types of two-qubit gates (e.g., CZ, iSWAP, and ECR) and single-qubit gates, we can obtain the quantum circuits in **b**, **c**, and **d**.

Supplementary Table 1. **Numerical results of QST with digital quantum gates.** We use IBM Qiskit [22] to simulate the noisy circuits for QST and calculate the transfer fidelity. Examples of quantum circuits for transferring single-excitation in a 1×6 chain are shown in Supplementary Figure 26. The parameters to build the error models, including qubit energy relaxation time T_1, pure dephasing time T_2, the mean Pauli errors of single-qubit gates (SQ) and two-qubit gates (iSWAP, ECR and CZ), are obtained from previous literature.

Source	Pauli errors	T_1<br>T_2	Gate length	1×6 single excitation<br>Fidelity $\mathcal{F}$<br>Transfer time t_{QST}	6×6 single excitation<br>Fidelity $\mathcal{F}$<br>Transfer time t_{QST}	6×6 Bell state<br>Fidelity $\mathcal{F}$<br>Transfer time t_{QST}	6×6 two excitations<br>Fidelity $\mathcal{F}$<br>Transfer time t_{QST}
This paper	-	142 us<br>19 us	Not applied	0.992<br>125 ns	0.902<br>250 ns	0.840<br>250 ns	0.737<br>250 ns
Nature 574, 505 (2019) [7]	SQ: 0.16e-2<br>iSWAP: 0.62e-2	16 us<br>30 us	SQ: 25 ns<br>iSWAP: 12 ns	0.978<br>60 ns	0.961<br>120 ns	0.955<br>108 ns	0.929<br>108 ns
Nature 614, 676 (2023) [20]	SQ: 0.1e-2<br>CZ: 0.6e-2	20 us<br>30 us	SQ: 34 ns<br>CZ: 25 ns	0.953<br>590 ns	0.909<br>1180 ns	0.897<br>1062 ns	0.838<br>1062 ns
Nature 618, 500 (2023) [21]	SQ: 6.75e-4<br>ECR: 1.15e-2	293 us<br>157 us	SQ: 50 ns<br>ECR: 612 ns	0.941<br>6620 ns	0.889<br>13240 ns	0.848<br>11916 ns	0.808<br>11916 ns

[1] M. Christandl, N. Datta, A. Ekert, and A. J. Landahl, Perfect state transfer in quantum spin networks, Phys. Rev. Lett. **92**, 187902 (2004).
[2] Y. Yao, L. Xiang, Z. Guo, Z. Bao, Y.-F. Yang, Z. Song, H. Shi, X. Zhu, F. Jin, J. Chen, S. Xu, Z. Zhu, F. Shen, N. Wang, C. Zhang, Y. Wu, Y. Zou, P. Zhang, H. Li, Z. Wang, C. Song, C. Cheng, R. Mondaini, H. Wang, J. Q. You, S.-Y. Zhu, L. Ying, and Q. Guo, Observation of many-body Fock space dynamics in two dimensions, Nature Physics **19**, 1459 (2023).
[3] X. Y. Jin, A. Kamal, A. P. Sears, T. Gudmundsen, D. Hover, J. Miloshi, R. Slattery, F. Yan, J. Yoder, T. P. Orlando, S. Gustavsson, and W. D. Oliver, Thermal and residual excited-state population in a 3D transmon qubit, Phys. Rev. Lett. **114**, 240501 (2015).
[4] X. Li, Y. Ma, J. Han, T. Chen, Y. Xu, W. Cai, H. Wang, Y. Song, Z.-Y. Xue, Z.-q. Yin, and L. Sun, Perfect quantum state transfer in a superconducting qubit chain with parametrically tunable couplings, Phys. Rev. Appl. **10**, 054009 (2018).
[5] E. Jeffrey, D. Sank, J. Y. Mutus, T. C. White, J. Kelly, R. Barends, Y. Chen, Z. Chen, B. Chiaro, A. Dunsworth, A. Megrant, P. J. J. O'Malley, C. Neill, P. Roushan, A. Vainsencher, J. Wenner, A. N. Cleland, and J. M. Martinis, Fast accurate state measurement with superconducting qubits, Phys. Rev. Lett. **112**, 190504 (2014).
[6] C. Keele and A. Kay, Combating the effects of disorder in quantum state transfer, Phys. Rev. A **105**, 032612 (2022).
[7] F. Arute, K. Arya, R. Babbush, D. Bacon, J. C. Bardin, R. Barends, R. Biswas, S. Boixo, F. G. S. L. Brandao, D. A. Buell, B. Burkett, Y. Chen, Z. Chen, B. Chiaro, R. Collins, W. Courtney, A. Dunsworth, E. Farhi, B. Foxen, A. Fowler, C. Gidney, M. Giustina, R. Graff, K. Guerin, S. Habegger, M. P. Harrigan, M. J. Hartmann, A. Ho, M. Hoffmann, T. Huang, T. S. Humble, S. V. Isakov, E. Jeffrey, Z. Jiang, D. Kafri, K. Kechedzhi, J. Kelly, P. V. Klimov, S. Knysh, A. Korotkov, F. Kostritsa, D. Landhuis, M. Lindmark, E. Lucero, D. Lyakh, S. Mandrà, J. R. McClean, M. McEwen, A. Megrant, X. Mi, K. Michielsen, M. Mohseni, J. Mutus, O. Naaman, M. Neeley, C. Neill, M. Y.

Niu, E. Ostby, A. Petukhov, J. C. Platt, C. Quintana, E. G. Rieffel, P. Roushan, N. C. Rubin, D. Sank, K. J. Satzinger, V. Smelyanskiy, K. J. Sung, M. D. Trevithick, A. Vainsencher, B. Villalonga, T. White, Z. J. Yao, P. Yeh, A. Zalcman, H. Neven, and J. M. Martinis, Quantum supremacy using a programmable superconducting processor, Nature **574**, 505 (2019).

[8] C. E. Porter, Statistical properties of spectra, Physics Today **16**, 26 (1963).

[9] V. Oganesyan and D. A. Huse, Localization of interacting fermions at high temperature, Phys. Rev. B **75**, 155111 (2007).

[10] Y. Y. Atas, E. Bogomolny, O. Giraud, and G. Roux, Distribution of the ratio of consecutive level spacings in random matrix ensembles, Phys. Rev. Lett. **110**, 084101 (2013).

[11] R. Mondaini, K. Mallayya, L. F. Santos, and M. Rigol, Comment on "Systematic construction of counterexamples to the eigenstate thermalization hypothesis", Phys. Rev. Lett. **121**, 038901 (2018).

[12] F. M. Izrailev, Simple models of quantum chaos: Spectrum and eigenfunctions, Physics Reports **196**, 299 (1990).

[13] V. Zelevinsky, B. Brown, N. Frazier, and M. Horoi, The nuclear shell model as a testing ground for many-body quantum chaos, Physics Reports **276**, 85 (1996).

[14] V. Khemani, A. Vishwanath, and D. A. Huse, Operator spreading and the emergence of dissipative hydrodynamics under unitary evolution with conservation laws, Phys. Rev. X **8**, 031057 (2018).

[15] N. Margolus and L. B. Levitin, The maximum speed of dynamical evolution, Physica D: Nonlinear Phenomena **120**, 188 (1998).

[16] L. Mandelstam and I. G. Tamm, The uncertainty relation between energy and time in non-relativistic quantum mechanics, J. Phys. USSR **9**, 249 (1944).

[17] S. Balay, S. Abhyankar, M. F. Adams, S. Benson, J. Brown, P. Brune, K. Buschelman, E. Constantinescu, L. Dalcin, A. Dener, V. Eijkhout, J. Faibussowitsch, W. D. Gropp, V. Hapla, T. Isaac, P. Jolivet, D. Karpeev, D. Kaushik, M. G. Knepley, F. Kong, S. Kruger, D. A. May, L. C. McInnes, R. T. Mills, L. Mitchell, T. Munson, J. E. Roman, K. Rupp, P. Sanan, J. Sarich, B. F. Smith, S. Zampini, H. Zhang, H. Zhang, and J. Zhang, *PETSc/TAO Users Manual*, Tech. Rep. ANL-21/39 - Revision 3.19 (Argonne National Laboratory, 2023).

[18] V. Hernandez, J. E. Roman, and V. Vidal, SLEPc: A scalable and flexible toolkit for the solution of eigenvalue problems, ACM Trans. Math. Software **31**, 351 (2005).

[19] J. R. McClean, S. Boixo, V. N. Smelyanskiy, R. Babbush, and H. Neven, Barren plateaus in quantum neural network training landscapes, Nature Communications **9**, 4812 (2018).

[20] R. Acharya, I. Aleiner, R. Allen, T. I. Andersen, M. Ansmann, F. Arute, K. Arya, A. Asfaw, J. Atalaya, R. Babbush, D. Bacon, J. C. Bardin, J. Basso, A. Bengtsson, S. Boixo, G. Bortoli, A. Bourassa, J. Bovaird, L. Brill, M. Broughton, B. B. Buckley, D. A. Buell, T. Burger, B. Burkett, N. Bushnell, Y. Chen, Z. Chen, B. Chiaro, J. Cogan, R. Collins, P. Conner, W. Courtney, A. L. Crook, B. Curtin, D. M. Debroy, A. Del Toro Barba, S. Demura, A. Dunsworth, D. Eppens, C. Erickson, L. Faoro, E. Farhi, R. Fatemi, L. Flores Burgos, E. Forati, A. G. Fowler, B. Foxen, W. Giang, C. Gidney, D. Gilboa, M. Giustina, A. Grajales Dau, J. A. Gross, S. Habegger, M. C. Hamilton, M. P. Harrigan, S. D. Harrington, O. Higgott, J. Hilton, M. Hoffmann, S. Hong, T. Huang, A. Huff, W. J. Huggins, L. B. Ioffe, S. V. Isakov, J. Iveland, E. Jeffrey, Z. Jiang, C. Jones, P. Juhas, D. Kafri, K. Kechedzhi, J. Kelly, T. Khattar, M. Khezri, M. Kieferová, S. Kim, A. Kitaev, P. V. Klimov, A. R. Klots, A. N. Korotkov, F. Kostritsa, J. M. Kreikebaum, D. Landhuis, P. Laptev, K.-M. Lau, L. Laws, J. Lee, K. Lee, B. J. Lester, A. Lill, W. Liu, A. Locharla, E. Lucero, F. D. Malone, J. Marshall, O. Martin, J. R. McClean, T. McCourt, M. McEwen, A. Megrant, B. Meurer Costa, X. Mi, K. C. Miao, M. Mohseni, S. Montazeri, A. Morvan, E. Mount, W. Mruczkiewicz, O. Naaman, M. Neeley, C. Neill, A. Nersisyan, H. Neven, M. Newman, J. H. Ng, A. Nguyen, M. Nguyen, M. Y. Niu, T. E. O'Brien, A. Opremcak, J. Platt, A. Petukhov, R. Potter, L. P. Pryadko, C. Quintana, P. Roushan, N. C. Rubin, N. Saei, D. Sank, K. Sankaragomathi, K. J. Satzinger, H. F. Schurkus, C. Schuster, M. J. Shearn, A. Shorter, V. Shvarts, J. Skruzny, V. Smelyanskiy, W. C. Smith, G. Sterling, D. Strain, M. Szalay, A. Torres, G. Vidal, B. Villalonga, C. Vollgraff Heidweiller, T. White, C. Xing, Z. J. Yao, P. Yeh, J. Yoo, G. Young, A. Zalcman, Y. Zhang, and N. Zhu, Suppressing quantum errors by scaling a surface code logical qubit, Nature **614**, 676–681 (2023).

[21] Y. Kim, A. Eddins, S. Anand, K. X. Wei, E. van den Berg, S. Rosenblatt, H. Nayfeh, Y. Wu, M. Zaletel, K. Temme, and A. Kandala, Evidence for the utility of quantum computing before fault tolerance, Nature **618**, 500–505 (2023).

[22] Qiskit contributors, Qiskit: An open-source framework for quantum computing (2023).
